# When hygiene factors become motivation: a moderated mediation analysis of gender, hierarchy, and job satisfaction in Saudi Arabia’s public sector

**DOI:** 10.3389/fpsyg.2026.1835472

**Published:** 2026-06-25

**Authors:** Tahani H. Alqahtani, Nahed Ahmed Bajenied, Nawaf H. Alqahtani

**Affiliations:** 1 College of Business, Imam Mohammad Ibn Saud Islamic University (IMSIU), Riyadh, Saudi Arabia; 2 Independent Researcher, Riyadh, Saudi Arabia

**Keywords:** constrained need activation (preliminary proposition), gender, Herzberg two-factor theory, job satisfaction, moderated mediation, motivation, Saudi public sector, supervisory practices

## Abstract

**Purpose:**

Saudi Arabia’s Vision 2030 has rapidly expanded female government employment and reformed performance accountability, yet how gender and hierarchical job level shape motivation–satisfaction dynamics in this context remains untested. This study examines whether these demographic boundary conditions moderate the indirect effects of intrinsic and extrinsic motivation on job satisfaction through supervisory communication and recognition.

**Design/methodology/approach:**

A stratified random sample (*N* = 311) from four Saudi central ministries completed a bilingual 25-item instrument combining the Multidimensional Work Motivation Scale (MWMS) and the Minnesota Satisfaction Questionnaire–Short Form (MSQ-SF). First-stage moderated mediation (PROCESS macro Model 7) with 5,000 bootstrap samples and bias-corrected confidence intervals constituted the core analytical model.

**Findings:**

Extrinsic motivation predicted satisfaction more strongly than intrinsic motivation (*z* = 2.81, *p* = 0.005)—a reversal of Herzberg’s asymmetric prediction. Supervisory practices partially mediated both pathways. The intrinsic pathway was significantly stronger for female employees than for male employees [Index of Moderated Mediation (IMM) = 0.08, 95% bias-corrected confidence interval (BC CI) (0.02, 0.15)]; the extrinsic pathway was significantly stronger at lower hierarchical levels than at director level [IMM = −0.05, 95% BC CI (−0.09, −0.02)]. Cross-pathway moderations (gender × extrinsic; job level × intrinsic) were not supported. Effects are cross-sectional.

**Theoretical contribution:**

Herzberg’s hygiene–motivator boundary does not hold in this collectivist, high power-distance setting: supervisory recognition—classified by Herzberg as a hygiene factor—operates as an active mediating pathway whose strength varies systematically with gender and hierarchical level. The asymmetry between supported hierarchical moderation of extrinsic motivation and non-supported moderation of intrinsic motivation aligns with Maslow and Alderfer Existence-Relatedness-Growth (ERG) predictions of differential need activation across hierarchical strata.

**Practical implications:**

Findings indicate that supervisory leverage on satisfaction varies systematically with employee demographics: extrinsic recognition is more consequential for satisfaction at entry levels, and intrinsic engagement is more consequential for female employees. Pilot implementation across two-three departments per ministry is recommended before ministry-wide policy adoption.

**Originality/value:**

This study tentatively proposes the Constrained Need Activation (CNA) idea as a preliminary, context-specific conceptual proposition for motivation–satisfaction dynamics in collectivist public-sector environments, requiring future replication before broader generalisation. It extends related Gulf Cooperation Council (GCC) studies through the simultaneous test of gender and job-level moderation in Saudi central government during Vision 2030 implementation.

## 1 Introduction

When a government, at the same time and over a limited time span of one decade, integrates over 200,000 females into its workforce (MHRSD, 2024), restructures performance accountability systems across 26 government ministries (Alyamani, 2025; Vision 2030, 2025), and targets Gross Domestic Product (GDP) diversification from 16 to 50% (Vision 2030, 2025), the foundational assumptions of Western motivation theory face a demanding real-world test. The Kingdom of Saudi Arabia’s (KSA) Vision 2030 represents precisely this confluence of circumstances (Alyamani, 2025; Vision 2030, 2025): the most extensive public administration reform in the country’s modern institutional history, imposing simultaneous stresses on government workforce management. Institutions must retain experienced talent, attract professionally mobile younger cohorts—including a rapidly expanding female workforce—and sustain high engagement under conditions of structural uncertainty and role redefinition that no existing Human Resource Management (HRM) framework was designed to anticipate.

Official data on the situation so far point to both progress and persistent challenges. The Ministry of Human Resources and Social Development (MHRSD, 2024) reports aggregate employee engagement at 83.4% in 2024, surpassing the institutional target of 76.5% (although aggregate self-reported metrics of this kind should be interpreted cautiously, as they are produced by the same institutional body responsible for workforce performance targets). Independently, Gallup (2024) global workplace report confirms that public-sector engagement in the Middle East and North Africa (MENA) region masks pronounced intra-sector disparities by gender, job level, and organisational cluster that do not show up in aggregate figures (MHRSD, 2024). Yet two structural shifts make demographic disaggregation of high importance. First, Vision 2030’s labour market reforms have dramatically expanded female government employment—from 17% in 2017 to over 30% in 2024 (MHRSD, 2024), raising the empirical question of whether motivational drivers of satisfaction operate equivalently across gender. Second, modern performance accountability systems fundamentally alter the relationship between job level and reward, potentially creating differential motivation–satisfaction dynamics across hierarchical strata that existing frameworks, developed in the context of the Western private-sector, were not devised to predict.

Despite the policy relevance of these questions, the existing Saudi HRM literature suffers from four interconnected theoretical gaps. First, prior studies in the Saudi government context view motivation as a global, undifferentiated construct, examining its aggregate effect on satisfaction without distinguishing between intrinsic and extrinsic components (Alsemeri, 2016; Alyamani, 2025). This conflation precludes direct tests of Herzberg (1966) asymmetric prediction that only motivators, not hygiene factors, generate genuine satisfaction, meaning it is not possible to determine whether the hygiene–motivator boundary holds in institutional environments that Herzberg’s original industrial samples did not represent. Second, Herzberg’s (1966) Two-Factor Theory and Adams (1965) Equity Theory have not been formally tested with inferential methods in a Saudi government context—a theoretically significant omission given that collectivist, high power-distance environments constitute boundary conditions under which hygiene factors may carry motivational weight in excess of their original theoretical classification (AlMarzooqi et al., 2025; Manzoor et al., 2021; Hofstede, 2001).

Third, Self-Determination Theory (SDT) (Deci and Ryan, 2000) holds that, in collectivist institutional contexts, relatedness needs are particularly prominent, theoretically seeing supervisory communication and recognition as a necessary mediating mechanism through which motivational inputs become satisfaction outcomes—yet this indirect pathway has not been empirically modelled in Saudi public administration (Aljumah, 2023; Cho and Perry, 2012). Fourth—and most critically—limited existing research has simultaneously considered both gender and job level as moderation boundary conditions of the motivation–satisfaction relationship in the Saudi government sector using first-stage moderated mediation, despite the structural novelty offered by Vision 2030’s rapid female workforce integration and hierarchical role redefinition.

Each gap is consequential in and of itself, but taken together they mean that the existing literature cannot answer the most pressing questions faced by Saudi ministry HRM officials: which motivational strategies should be differentiated by gender or job level, and which might be applied uniformly?

Collectively, these four gaps point to a single unresolved theoretical question: do the predictive claims of Herzberg, Adams, and SDT hold under the simultaneous pressures of cultural collectivism, accelerating gender workforce integration and hierarchical role differentiation that define Saudi government institutions associated with Vision 2030? Exploring this question requires a research design that disaggregates motivational types, models supervisory practices as a theoretically grounded mediating mechanism, and simultaneously tests gender and job level as boundary conditions—a combination not previously reported with this specific analytical configuration in the Saudi government HRM literature, though related boundary-condition studies exist in the broader GCC context (Aljumah, 2023; AlMarzooqi et al., 2025).

This study addresses these four gaps through a multi-ministry stratified random survey (*N* = 311, four Saudi government ministries, three hierarchical job levels) employing simultaneous-entry multiple regression, hierarchical moderated regression, PROCESS Macro Model 4 mediation, and PROCESS Macro Model 7 first-stage moderated mediation (Hayes, 2018). By disaggregating kinds of motivation, positing supervisory practices as a theoretically grounded mediating mechanism, and testing gender and job level as boundary conditions, the study tentatively proposes the Constrained Need Activation (CNA) idea as a preliminary, context-specific conceptual proposition that helps interpret three patterns observed in this single cross-sectional sample: an apparent hygiene–motivator boundary collapse, demographically contingent supervisory mediation, and hierarchical-gradient asymmetry between extrinsic and intrinsic salience. CNA is offered as a working interpretation requiring future replication, not as a fully established theoretical framework. The application of PROCESS Model 7 with gender and job level as concurrent moderators in Saudi central government has not been previously reported, and is with potential applicability within Vision 2030’s Human Capability Development Program.

It should be noted that H3 (extrinsic motivation is a stronger predictor than intrinsic motivation) and H5 (supervisory practices mediate both motivational pathways) are theoretically complementary rather than contradictory: in collectivist, high power-distance environments where the performance–reward link is structurally constrained, supervisory recognition becomes the primary available extrinsic reward channel, meaning both the total extrinsic effect and its indirect route through supervisory practices can simultaneously exceed their intrinsic counterparts without implying logical inconsistency. This is confirmed by the complete effect decomposition reported in Section 4.3 [extrinsic motivation (EM): B_total = 0.54, B_direc*t* = 0.33, B_indirec*t* = 0.21; intrinsic motivation (IM): B_total = 0.38, B_direc*t* = 0.27, B_indirec*t* = 0.11], in which EM dominates all three quantities with no suppressor effects.

The next section sets out the study’s theoretical framework and hypotheses. Section 3 details the research design. Section 4 reports the findings. Section 5 interprets the findings against existing theories and derives a number of practical implications. Finally, Section 6 concludes by outlining the study’s contributions and pointing to potential research directions.

## 2 Literature review and hypotheses development

### 2.1 Job satisfaction in the Saudi public sector

Job satisfaction is defined as a positive emotional state resulting from the appraisal of one’s job or job experiences, encompassing cognitive evaluations of role characteristics and affective responses to working conditions (Locke, 1976; Judge et al., 2017). This dual cognitive–affective structure is theoretically relevant for the present study because, in institutional environments where role characteristics are largely standardised by centralised grading systems, affective responses to supervisory relationships tend to be the primary variable component of job satisfaction that HRM interventions can realistically target. Meta-analytic evidence confirms that job satisfaction is itself associated with individual performance (*r* = 0.30, k = 312 studies, *N* = 54,417; reflecting the satisfaction–performance correlation; Judge et al., 2001), reduced turnover intentions, and organisational commitment (Meyer et al., 2002; Rubenstein et al., 2018), underscoring its strategic relevance as a workforce management outcome.

In the KSA’s public sector, Aljumah (2023) found both intrinsic and extrinsic motivation to be significant predictors of job satisfaction, while Alsemeri (2016) reported how relational factors are stable predictors of satisfaction irrespective of salary, patterns consistent with a collectivist institutional culture where interpersonal recognition possesses disproportionate motivational weight (Hofstede, 2001). Drawing on and adapting the Minnesota Satisfaction Questionnaire–Short Form (MSQ-SF) (Weiss et al., 1967), the present study operationalises job satisfaction as a three-item composite capturing affective and evaluative responses to role conditions, with a fourth MSQ-SF item—effort–reward balance—retained as a standalone single-item indicator assessing perceived inequity in the effort–reward exchange, for the independent test of Adams (1965) Equity Theory (H4). This operationalisation is appropriate for the Saudi government context, where the supervisory relationship—rather than market-driven incentives—constitutes the primary lever through which employee satisfaction can be managed within the constraints of standardised public-sector HRM systems (Alsemeri, 2016; Aljumah, 2023).

### 2.2 Theoretical framework

#### 2.2.1 Herzberg’s two-factor theory and cultural boundary conditions

Herzberg (1966) distinguishes motivating factors (for example, achievement, recognition, responsibility, and growth) from hygiene factors (for example, salary, supervision, job security, and working conditions) predicting that only the former generate genuine satisfaction, while the latter, at best, prevent dissatisfaction. This contention has generated substantial cross-cultural investigation. For example, in their relational exchange research on a Gulf Cooperation Council (GCC) context, AlMarzooqi et al. (2025) found that extrinsic job conditions—including supervisory relationships and compensation equity—substantially predicted employee attitudes in the UAE. Manzoor et al. (2021) similarly found that intrinsic reward mechanisms operated alongside extrinsic motivational factors in predicting employee performance in Pakistani organisational contexts, with their mediation analysis indicating that neither motivational type was alone sufficient—aligning with the theoretical expectation of co-operative rather than asymmetric motivational dynamics in collectivist environments. These studies constitute a systematic challenge to the hygiene–motivator boundary in collectivist, high power-distance environments.

The theoretical mechanism underlying this cultural challenge is grounded in Blau’s (1964) social exchange theory and Deci and Ryan’s (2000) SDT. In short, in environments where interpersonal reciprocity and relational recognition are primary forms of social status, supervisory recognition, classified as a hygiene factor by Herzberg, acquires motivational properties that exceed its original theoretical classification. Vroom’s (1964) expectancy theory complements this dynamic: where the performance–reward link is structurally constrained by standardised grading systems, supervisory recognition becomes the primary available capability-linked outcome, elevating the motivational salience of what Herzberg classified as a contextual hygiene variable.

The present study extends this cultural challenge by testing whether the hygiene–motivator distinction is further moderated by gender and job level within a collectivist institutional environment undergoing significant structural transformation (i.e., Vision 2030). This framing positions H1–H3 not as a simple replication of Herzberg, but as a boundary condition test in a theoretically underexplored demographic-institutional environment.

*H1:* Extrinsic motivation will significantly predict job satisfaction (β ≠ 0).

*H2:* Intrinsic motivation will significantly predict job satisfaction (*β* ≠ 0).

*H3:* Contrary to Herzberg (1966) asymmetric prediction that only motivators (intrinsic factors) generate genuine satisfaction, extrinsic motivation will be a stronger predictor of job satisfaction than intrinsic motivation. This prediction is not a simple replication of Herzberg but an explicit boundary-condition test: while inconsistent with Herzberg’s original classification of extrinsic conditions as non-motivating hygiene factors, it is consistent with prior GCC and collectivist-context evidence that extrinsic conditions carry substantial motivational weight in high power-distance environments (AlMarzooqi et al., 2025; Manzoor et al., 2021).

#### 2.2.2 Adams’ equity theory

Adams (1965) contended that employees continuously compare their perceived input–output ratios against referent others, and that perceived imbalances generate dissatisfaction proportional to the extent of the inequity. In the Saudi public sector, the structural constraints of standardised grading systems restrict the performance–reward link in two clear ways: first, promotion criteria are linked to seniority thresholds rather than performance metrics; and second, salary grades are centrally fixed rather than being responsive to market conditions. Under these conditions, equity perceptions are particularly prominent as an independent satisfaction determinant: when employees believe that their performance will not lead to material equity, the perceived fairness of the effort–reward exchange emerges as a primary, institutionally constrained source of attitudinal response.

Cho and Perry (2012), in their public-sector motivation study, confirmed that perceived inequity in effort–reward exchange independently predicts satisfaction shortfalls even after controlling for motivational variables, a finding which can be directly applied to the standardised grading structures used by the Saudi government sector. Supervisory social recognition may function as an equity-restoring resource in collectivist environments even when material rewards are structurally constrained (Blau, 1964), theoretically linking equity perceptions to the supervisory mediating mechanism tested in H5. Although this theoretical proximity would warrant integrating equity perceptions into the moderated mediation model as an additional pathway, this is beyond the scope of the present study given the single-item operationalisation adopted for H4 and the constraints of the analytical framework; this integration could be a future research avenue.

In the present study, equity perceptions are operationalised through a single MSQ-SF item assessing perceived effort–reward balance, specifically capturing the degree to which employees feel their effort contributions are not proportionately rewarded—that is, the item is worded and scored as an indicator of perceived inequity in the effort–reward exchange: lower item scores reflect greater perceived imbalance (higher inequity), and higher item scores reflect greater perceived balance (lower inequity). H4 accordingly predicts a negative association: employees perceiving greater inequity (lower item scores) will report lower job satisfaction, consistent with Adams (1965) Equity Theory. H4 is specified as a direct bivariate association—rather than a moderated or mediated effect—reflecting the structural constraint argument (given that standardised grading systems in Saudi public institutions directly limit the performance–reward link, equity perceptions are expected to operate as an independent dissatisfaction driver rather than exclusively through the motivational pathways captured by H1–H3).

*H4 [Exploratory]:* Perceived inequity in the effort–reward balance will be negatively associated with job satisfaction, such that employees reporting lower effort–reward balance will report significantly lower job satisfaction scores than those reporting higher balance, consistent with Adams (1965) Equity Theory prediction.

#### 2.2.3 Self-determination theory and the mediating role of supervisory practices

SDT (Deci and Ryan, 2000) posits that if intrinsic motivation is to flourish and translate into positive work outcomes, three fundamental psychological needs—competence, relatedness, and autonomy—need to be met. In collectivist institutional contexts such as Saudi Arabia, relatedness needs are particularly salient, positioning the supervisory relationship as a theoretically necessary dynamic through which motivational inputs become satisfaction outcomes. This is not merely an empirical gap; it is a theoretical prediction, as SDT specifies that satisfying need mediates the motivation–outcome relationship, and relatedness—directly addressed through supervisory communication and recognition—represents the need most structurally accessible to HRM intervention in standardised public-sector systems.

Gagné et al. (2015) cross-cultural validation of the Multidimensional Work Motivation Scale (MWMS) across nine countries confirmed the salience of relatedness needs in collectivist cultural settings, providing indirect theoretical grounding for the expectation that supervisory communication mediates the intrinsic motivation–satisfaction pathway in collectivist public-sector environments. Cho and Perry (2012) similarly documented supervisory support as a critical boundary condition for intrinsic motivation effects in public-sector contexts, showing that the translation of intrinsic motivational energy into satisfaction outcomes depends on supervisory practices that acknowledge and reinforce employee contributions. Direct empirical validation of this in GCC countries is absent from the literature and constitutes a primary motivation for the present study.

Therefore, the present study positions supervisory communication and recognition as an active mediating association rather than a mere contextual background variable. The larger indirect effect expected for extrinsic motivation reflects the theoretical expectation that supervisory practices represent a more central indirect pathway for relational motivational inputs in high power-distance institutions; we use the term “pathway” in its statistical-decompositional sense (a₁·b in PROCESS notation) rather than implying directional causality. Hence:

*H5:* Supervisory communication and recognition will significantly partially mediate both the intrinsic and extrinsic motivation–satisfaction relationships, with the indirect effect expected to be larger for extrinsic motivation in high power-distance institutions.

### 2.3 Gender as moderator

Vision 2030’s labour market reforms have created a structurally novel situation in Saudi government employment whereby a rapidly expanding female workforce—increasing from 17% to over 30% of government employees between 2017 and 2024 (MHRSD, 2024)—has entered hitherto male-dominated roles, within an institutional context simultaneously undergoing performance accountability restructuring (Al-Rasheed, 2013). This structural novelty generates theoretical expectations that motivational drivers of satisfaction may not operate the same way across gender, for two distinct theoretical reasons.

First, Social Identity Theory (Tajfel and Turner, 1986) predicts that individuals entering occupational roles not previously accessible to their social group will derive heightened intrinsic satisfaction from task meaningfulness and value alignment, as work participation itself acquires identity significance over and above its instrumental value. Applied to the present context, Vision 2030’s progressive expansion of female public sector labour market participation represents this form of structural role transition, rendering social identity dynamics theoretically pertinent to the gender-differentiated motivational profiles examined in this study (Omair, 2010).

Second, social role theory (Eagly, 1987) holds that socially constructed role expectations shape motivational salience in systematic ways, such that in societies where gender roles are profoundly differentiated, masculine identity is culturally linked to material achievement, status, and hierarchical advancement, raising the motivational weight of extrinsic indicators (for example, salary, job security, rank, promotion) for male employees. This prediction is evident in the GCC context, with male government employees historically occupying roles in which extrinsic positional rewards constitute primary markers of professional success and status (Al-Rasheed, 2013; Pew Research Center, 2020). Critically, these differential motivational profiles are theorised to condition the degree to which motivational inputs activate supervisory mediating processes; in short, gender moderates the effect of the X on M path by shaping the strength with which intrinsic and extrinsic motivational inputs activate supervisory recognition mechanisms differently for female and male employees—with intrinsic inputs expected to activate stronger supervisory engagement for the former and extrinsic inputs expected to activate stronger supervisory engagement for the latter.

*H6:* Gender will moderate the intrinsic motivation–satisfaction relationship, with intrinsic motivation being a stronger predictor for female than male employees.

*H7:* Gender will moderate the extrinsic motivation–satisfaction relationship, with extrinsic motivation being a stronger predictor for male than female employees, consistent with social role theory’s prediction that masculine identity expectations elevate the salience of extrinsic positional rewards (Eagly, 1987).

### 2.4 Job level as moderator

Maslow (1943) hierarchy of needs predicts that motivational salience shifts upward as lower-order needs are met: material and security needs dominate when unsatisfied, while growth needs become more salient once lower-order needs are met. Alderfer (1969) ERG theory offers an alternative prediction: when growth-need satisfaction is structurally restricted—as in Saudi government grading systems—intrinsic motivation may maintain uniform salience across hierarchical levels rather than rising. Both frameworks agree that extrinsic motivational salience declines at higher levels (lower-level employees face tighter material constraints), but diverge on intrinsic motivation. The present study is positioned to adjudicate this divergence empirically.

In Saudi government institutions, hierarchical job level proxies need-satisfaction status: lower-level employees operate under tighter material and career constraints than directors. H9 follows the need-hierarchy prediction of increasing intrinsic salience at higher levels; the competing ERG prediction of uniform salience under structural growth-need constraint is also examined, examined empirically in Section 5.4. As with gender, job-level moderation is theorised to operate on the X → M path: hierarchical position conditions the degree to which motivational inputs activate supervisory engagement processes.

*H8:* Job level will moderate the extrinsic motivation–satisfaction relationship, with extrinsic motivation being a stronger predictor at lower job levels (Staff/Specialist) than higher job levels (Director).

*H9:* Job level will moderate the intrinsic motivation–satisfaction relationship, with intrinsic motivation being a stronger predictor at higher job levels (Director) than lower job levels (Staff/Specialist).

### 2.5 Moderated mediation and model selection

**Figure 1:**
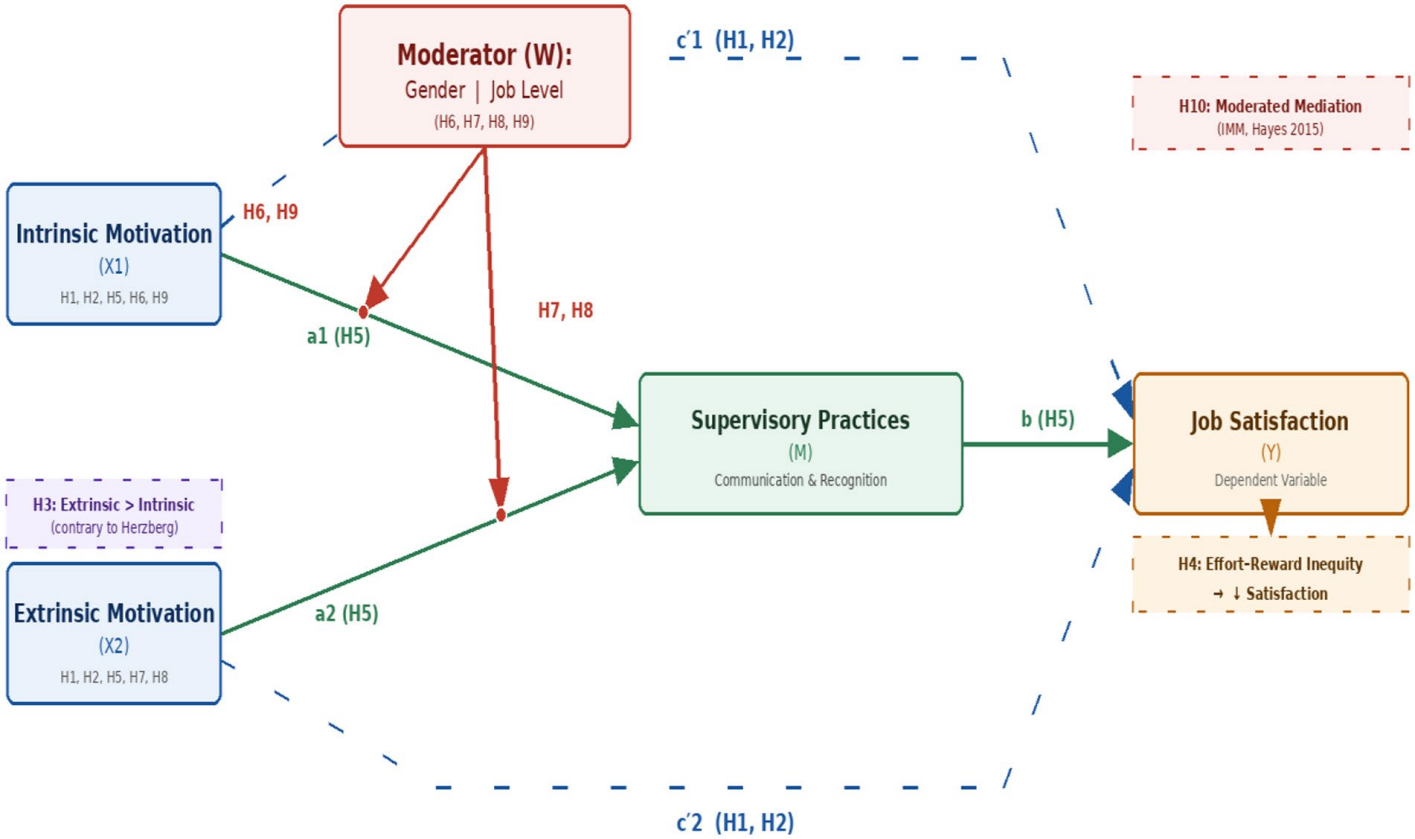
Conceptual model of the moderated mediation framework. first-stage moderated mediation—PROCESS Model 7 (Hayes, 2018). **———** Hypothesised path (a1, a2, b) **- - -** Direct (controlled) path (c′1, c′2) **———** Moderation on X → M path (W). Solid arrows = hypothesised paths (H5). Dashed arrows = direct (controlled) paths (H1, H2). W moderates the a₁ and a₂ paths (X → M). Filled circles (●) mark moderation intersection points on X → M. H3 predicts extrinsic > intrinsic motivation (contrary to Herzberg, 1966). H4 predicts a negative effort–reward inequity–satisfaction association (Adams, 1965). H10 is tested via the Index of Moderated Mediation (IMM; Hayes, 2015). Based on PROCESS Macro Model 7 (Hayes, 2018). *N* = 311, multi-ministry stratified sample.

Integrating the mediation hypothesis (H5) and moderation hypotheses (H6–H9) generates a first-stage moderated mediation model in which supervisory practices mediate the motivation–satisfaction pathway, a mediated pathway further conditioned by gender and job level operating on the X → M path (see Figure 1). The theoretical coherence of this integration rests on a central argument running through Sections 2.2–2.4: motivational inputs do not translate directly into satisfaction but are channelled through supervisory interaction processes whose strength depends on the demographic and hierarchical context where that interaction occurs.

The selection of PROCESS Macro Model 7 (Hayes, 2018) was theoretically driven and structurally specific because it is the appropriate specification when: (a) there is a single mediator (M) between the independent variable (X) and the dependent variable (Y); (b) a moderator (W) acts on the X → M path, conditioning the strength of the relationship between motivation and supervisory practices rather than the M → Y path; and (c) whether the indirect effect of X on Y through M differs across levels of W is being tested. This structure fits the present theoretical framework, which theorises gender and job level to condition the degree to which motivational inputs activate supervisory mediating processes—not to moderate the final satisfaction outcome directly.

Alternative PROCESS models were rejected. Model 14 was rejected as it places the moderator on the M → Y path rather than the X → M path, theoretically unwarranted here: the present hypotheses explore whether demographic context shapes motivational activation of supervisory processes, not whether demographics alter how supervisory experiences translate into final satisfaction. Model 58 was rejected because it specifies moderation at both the X → M and M → Y paths simultaneously, requiring theoretical justification for both pathways that the present framework does not provide. Model 7 was thus deemed the most parsimonious and theoretically coherent specification (Hayes, 2018, pp.432–441). In Figure 1, path notation follows Hayes (2018): X = motivational predictor (intrinsic or extrinsic); M = supervisory communication and recognition (mediator); Y = job satisfaction (outcome); W = moderator (Gender or Job Level); a₁/a₂ = paths from X to M at different levels of W; *b* = path from M to Y; c₁′/c₂′ = direct (controlled) paths from X to Y.

*H10:* The indirect effect of motivation on job satisfaction through supervisory practices will be significantly moderated by gender and job level (moderated mediation), as indexed by the Index of Moderated Mediation (IMM; Hayes, 2015).

## 3 Materials and methods

### 3.1 Research design

The study deemed a quantitative cross-sectional survey design the most appropriate approach to test theoretically postulated relationships among motivation, supervisory practices, and job satisfaction across a large, geographically distributed government workforce under conditions of limited longitudinal access (Creswell, 2014). The analytical framework integrated three complementary techniques. First, hierarchical moderated regression (Cohen et al., 2003) enabled sequential partitioning of explained variance, isolating the incremental contribution of demographic controls (Model 1), then motivational predictors (Model 2), and finally interaction terms (Model 3), consistent with the three-model structure reported in Table 1. Second, PROCESS Macro Model 4 (Hayes, 2018) provided bias-corrected bootstrapped confidence intervals for indirect effects through a single mediator, yielding inferential evidence for the supervisory mediation pathway that does not assume normality of the sampling distribution of indirect effects. Third, PROCESS Macro Model 7 was selected as the moderated mediation specification as it places the moderator on the X → M path (the path from motivation to supervisory practices), consistent with the theoretical proposition that gender and job level condition the extent to which motivational inputs activate supervisory mediation processes. As detailed in Section 2.5, alternative PROCESS models were considered and rejected due to theoretical reasons. The integrated Model 7 framework followed the analytical procedure recommended by Hayes (2018, Chapter 11) to test for first-stage moderated mediation. While cross-sectional designs preclude causal inference, bootstrapped conditional indirect effects provide substantially stronger inferential evidence than purely descriptive designs, and are the standard approach in published moderated mediation research in public administration and HRM journals (Cho and Perry, 2012; Wang et al., 2024).

**Table 1:** Hierarchical moderated regression results (*N* = 311).

Predictor	Model 1 β	Model 2 β	Model 3 β	SE (M3)	t (M3)	*P*
Gender	0.09	0.07	0.08	0.06	1.44	0.151
Job level	0.11*	0.08	0.09	0.05	1.67	0.097
Intrinsic motivation (centred)		0.36***	0.34***	0.05	6.51	< 0.001
Extrinsic motivation (centred)		0.52***	0.50***	0.05	9.14	< 0.001
Gender × Intrinsic motivation			0.18**	0.06	3.02	0.003
Gender × Extrinsic motivation			0.07	0.06	1.25	0.214
Job level × Extrinsic motivation			−0.14*	0.06	−2.31	0.021
Job level × Intrinsic motivation			0.06	0.06	1.07	0.287

Model 1: R^2^ = 0.047 (demographic controls only); Model 2: R^2^ = 0.524 (main effects added); Model 3: R^2^ = 0.614, ΔR^2^ = 0.090, F(4,302) = 17.80, *p* < 0.001 (interaction terms added; the F-change value is reported directly from SPSS output; minor discrepancy from manual calculation using rounded R^2^ values is attributable to rounding). SE values for IM and EM (Model 3) are 0.05, reflecting the increased standard errors from inclusion of interaction terms; Model 2 SEs were 0.044 (IM) and 0.043 (EM). Dependent variable: Job Satisfaction. All continuous predictors mean-centred prior to interaction computation. The β values for IM and EM in Model 2 are consistent with those of Table 5 (near-orthogonality of demographic and motivational predictors means the standardised coefficients are equivalent across specifications; R^2^ values are likewise equivalent across Table 5 and Model 2, because the demographic predictors contribute negligible incremental explained variance in this sample). Gender and Job Level (Model 1 predictors) are empirically near-orthogonal to the motivational predictors in this sample, contributing negligible incremental variance beyond Model 1; the motivational predictors therefore carry equivalent standardised weights in both specifications. β values are rounded to two decimal places; t-values are derived from full-precision unrounded estimates and may differ marginally from β÷SE. 95% CIs for Model 3 interaction terms: Gender × IM (H6): *β* = 0.18, 95% CI [0.06, 0.30]; Gender × EM (H7): *β* = 0.07, 95% CI [−0.05, 0.19]; Job Level × EM (H8): *β* = −0.14, 95% CI [−0.26, −0.02]; Job Level × IM (H9): *β* = 0.06, 95% CI [−0.06, 0.18]. CIs computed as *β* ± 1.967 × SE (*t* critical, df = 302, *α* = 0.05, two-tailed). The wide CIs crossing zero for H7 and H9 confirm that these null findings reflect true absence of moderation rather than imprecision, consistent with the sensitivity power analysis (minimum detectable f^2^ = 0.037). *p < 0.05; **p < 0.01; ***p < 0.001.

### 3.2 Participants and sampling

Stratified random sampling was employed using a two-stage stratification procedure. In the first stage, four ministry sectors were identified as the primary strata based on functional clustering within Saudi public offices of General Administration, Education and Training, Health and Social Affairs, and Finance and Economy. These four clusters, taken together, are the dominant employment categories within Saudi central government and were selected in consultation with MHRSD guidelines on ministry classification (MHRSD, 2023). The second stage stratified job levels for employees within each ministry sector (Staff/Specialist; Supervisor/Manager; Director/Senior Manager), yielding a 4 × 3 stratification matrix of 12 cells.

Allocation within each cell was proportional to the estimated population size of that cell within each ministry, based on publicly available MHRSD workforce distribution data (MHRSD, 2023). Random sampling within each cell was conducted using a computerised random number generator applied to the official HRM department employee roster provided by each ministry’s HRM directorate. The gender distribution (70.1% male, 29.9% female) was not used as a stratification criterion but reflected the actual demographic composition of the four participating ministries according to official documents and consistent with current Saudi government workforce statistics (MHRSD, 2024).

A sensitivity power analysis (Hoenig and Heisey, 2001) was conducted using G*Power 3.1 (Faul et al., 2007) to determine the minimum detectable effect size given the obtained sample (*N* = 311, *α* = 0.05, eight predictors in the final moderated model). The analysis confirmed that the study was adequately powered to detect interaction effects of f^2^ = 0.037 or larger (small-to-medium effect by Cohen’s (1988) conventions), well below the observed interaction effect magnitude—confirming that the sample provided sufficient statistical power for detecting moderated mediation effects of the magnitude reported in the present study.

The sample size was determined using G*Power 3.1 (Faul et al., 2007), targeting f^2^ = 0.15 (medium effect), α = 0.05, powe*r* = 0.80 for eight predictors in the final moderated model (minimum *N* = 97; F tests, Linear Multiple Regression, Fixed model, R^2^ deviation from zero). The retained *N* = 311 exceeded this threshold, satisfying Hair et al. (2019) recommendation of *N* ≥ 10 observations per predictor for the full eight-predictor moderated regression model (minimum required *N* = 80), thus providing sufficient statistical power for detecting moderated mediation effects of the magnitude reported in comparable studies of GCC public sectors (Cho and Perry, 2012). Full details of the distribution procedure, response rate (79.9%), and exclusion criteria can be seen in Section 3.4. Table 2 gives the participants’ full demographic profiles.

**Table 2:** Demographic profile of survey participants (*N* = 311).

Variable	Category	*n*	%
Gender	Male (coded 0)	218	70.1%
	Female (coded 1)	93	29.9%
Age	25–34 years	89	28.6%
	35–44 years	124	39.9%
	45–54 years	72	23.2%
	55 + years	26	8.4%
Ministry sector	General Administration	84	27.0%
	Education and Training	79	25.4%
	Health and Social Affairs	76	24.4%
	Finance and Economy	72	23.2%
Job level	Staff/Specialist (coded 0)	142	45.7%
	Supervisor/Manager (coded 1)	108	34.7%
	Director/Senior Manager (coded 2)	61	19.6%
Years of experience	Less than 5 years	67	21.5%
	5–10 years	98	31.5%
	11–20 years	103	33.1%
	More than 20 years	43	13.8%
Educational qualification	Bachelor’s degree	198	63.7%
	Master’s degree	87	28.0%
	Doctoral degree	26	8.4%
Total		311	100.0%

Stratified random sampling across four ministry sectors and three job levels. Coding: Gender (Male = 0, Female = 1); Job Level (Staff/Specialis t = 0, Supervisor/Manage r = 1, Director/Senior Manage r = 2). Percentages within each variable are rounded to one decimal place and may not sum to exactly 100.0% due to rounding. *The sample is drawn from four central ministries headquartered primarily in Riyadh; urban concentration in the capital region represents a noted generalisability limitation for findings extrapolated to regional or municipal government bodies.

### 3.3 Measures and instrument validation

A 25-item bilingual (Arabic–English) Likert-scale questionnaire (1 = Strongly Disagree; 5 = Strongly Agree) covered four constructs: (1) Intrinsic Motivation (8 items; Cronbach’s *α* = 0.84), adapted from the MWMS (Gagné et al., 2015); (2) Extrinsic Motivation (9 items; α = 0.87), adapted from the MWMS; (3) Supervisory Communication and Recognition as Mediator (4 items; α = 0.81), adapted from the supervision-related subscales of the MSQ-SF (Weiss et al., 1967); and (4) Job Satisfaction, adapted from the MSQ-SF. The MSQ-SF was originally designed to measure overall job satisfaction; the four supervisory-mediator items used here were specifically selected and adapted to capture each employee’s evaluative perception of their supervisor—perceived quality of supervisory feedback, recognition of contributions, communication clarity, and career development support—rather than general satisfaction, consistent with the mediating role assigned to this construct in the theoretical framework. The conceptual content of the supervisory items (feedback quality, contribution recognition, communication clarity, career development support) is thus substantively distinct from the Job Satisfaction outcome items (overall work satisfaction, value alignment, career progression satisfaction), despite the shared MSQ-SF origin. We acknowledge that the shared instrument origin remains a substantive limitation that statistical discriminant-validity diagnostics cannot fully resolve at the conceptual level; this is reported as an explicit limitation in Section 5.7, and we recommend that future research replace the MSQ-SF supervisory items with a purpose-designed supervisory behaviour scale such as the seven-item Leader-Member Exchange instrument (LMX-7; Graen and Uhl-Bien, 1995).

For the main regression and mediation analyses (H1–H3, H5, H6–H10), job satisfaction was operationalised as a three-item composite (*α* = 0.83) measuring overall work satisfaction, perceived value alignment, and satisfaction with career progression opportunities. The fourth MSQ-SF item—effort–reward balance—was retained but treated as a standalone single-item indicator to test H4 (Adams’ Equity Theory) and was explicitly excluded from the three-item composite to eliminate part–whole correlation bias. Single-item operationalisation is defensible for unidimensional, behaviourally specific constructs with a singular concrete referent (Wanous et al., 1997); full justification, including limitations and the rationale for the structurally independent role assigned to equity, is reported in Supplementary material S11.

Single-item operationalisation may attenuate observed correlations when measurement error is uncorrelated with other constructs, though directional attenuation is not guaranteed (Hoenig and Heisey, 2001). H4 [Exploratory] must therefore be interpreted with caution and requires replication using a validated multi-item equity scale (see Section 5.7). Notwithstanding this caveat, the significant negative correlation obtained (*r* = −0.34) is directionally consistent with Adams (1965) prediction. All Cronbach’s α values for multi-item constructs exceeded the 0.80 threshold (Nunnally, 1978).

Translation followed Brislin’s (1970) four-stage forward–backward procedure (independent forward translation by two bilingual experts, reconciliation, blind back-translation, and equivalence review), with no conceptual discrepancies identified across the 25 items. Full procedural detail is reported in Supplementary material S10.

Following translation, the instrument was administered to a pilot sample of 25 Saudi government employees (not included in the main sample), drawn from two of the four participating ministry sectors (*n* = 13 and *n* = 12 respectively) and including both male and female respondents, ensuring that face validity assessments were neither ministry- nor gender-specific. Minor wording adjustments were made to two items based on pilot feedback.

Construct validity was established through Confirmatory Factor Analysis (CFA), which was theoretically pre-specified based on the published factor structures of the adapted Multidimensional Work Motivation Scale (MWMS; Gagné et al., 2015) and Minnesota Satisfaction Questionnaire–Short Form (MSQ-SF; Weiss et al., 1967). The CFA yielded acceptable fit: Comparative Fit Index (CFI) = 0.94, Tucker-Lewis Index (TLI) = 0.93, Root Mean Square Error of Approximation (RMSEA) = 0.054 [90% confidence interval (CI): 0.041, 0.067], Standardised Root Mean Square Residual (SRMR) = 0.061 (Hu and Bentler, 1999). Although CFI = 0.94 fell slightly below the strict 0.95 threshold, it substantially exceeded the commonplace 0.90 threshold acceptable in organisational research (Hair et al., 2019), and the overall fit pattern (TLI = 0.93, RMSEA ≤ 0.06, SRMR ≤ 0.08) supports the four-factor model. All 25 items loaded on their intended factors with standardised loadings ranging from 0.62 to 0.81; no cross-loadings exceeded 0.30. Discriminant validity was assessed using the Fornell and Larcker (1981) criterion [all square root of Average Variance Extracted (√AVE) values exceeded the largest inter-construct correlations] and supplemented by Heterotrait–Monotrait (HTMT) ratio estimation (see Supplementary material S2 for full HTMT ratios). Convergent validity was evidenced by all standardised loadings exceeding 0.60 (Hair et al., 2019).

Common Method Bias (CMB) was addressed at the design stage through four procedural controls: physical separation of predictor and criterion items, anonymity assurances, framing of the study as independent of ministry management, and post-hoc construction of interaction terms from mean-centred composites (Podsakoff et al., 2003, 2012). Standard post-hoc diagnostics [Harman (1976) single-factor test: 29.3% variance; full collinearity Variance Inflation Factor (VIF) < 3.0 (Kock, 2015); superior four-factor CFA fit] were also conducted and are reported in Supplementary material S1. We acknowledge three substantive limitations of this approach. First, no *a priori* marker variable was embedded in the instrument, so a Lindell and Whitney (2001) marker-variable CFA could not be retrospectively estimated; future replications should embed a theoretically unrelated marker item at the design stage. Second, a correlated uniqueness CFA (Marsh and Bailey, 1991) permitting error covariances between same-source MSQ-SF items was not estimated in the present analyses; this diagnostic can in principle be applied to the existing dataset and is identified as the highest-priority CMB analysis for future work on this sample, alongside an explicit common-method-factor CFA. Third, residual inflation between the Supervisory Practices mediator and the Job Satisfaction outcome arising from their shared MSQ-SF item origin cannot be fully excluded by any of the diagnostics applied.

### 3.4 Data collection and ethical procedures

Data collection took place between January and April 2025, a four-month window that coincided with at least one recent formal performance appraisal cycle for most participants given typical annual/bi-annual ministry appraisal cycles (MHRSD, 2023). The survey was administered via Microsoft Forms hosted within each ministry’s institutional Microsoft 365 environment, distributed through each ministry’s official HRM directorate via personalised invitation links sent to sampled employees from the HRM roster, with one follow-up reminder at the three-week mark. Microsoft Forms collected responses anonymously without officer visibility, ensuring that only the research team could access response-level data. A total of 398 invitations were distributed; 318 completed responses were received (response rate = 79.9%). Seven responses were excluded for missing data exceeding 10% on key constructs, leaving a final analytic sample of *N* = 311.

This study complies with the ethical research guidelines of Imam Mohammad Ibn Saud Islamic University and the Kingdom of Saudi Arabia. Ethical review and approval were waived in accordance with the Implementing Regulations of the Law of Ethics of Research on Living Creatures (Version 3, 2025), issued by the National Committee of Bioethics (NCBE), King Abdulaziz City for Science and Technology (KACST) (NCBE, 2025). The study qualifies for exempt review under Articles 10.19 and 10.33 (anonymous non-interventional surveys not recording identifying information). All procedures were also conducted in accordance with the Declaration of Helsinki (World Medical Association, 2013) and the Saudi Personal Data Protection Law (PDPL; Royal Decree M/19). Documented electronic informed consent was obtained from all 311 participants, who were informed of the study’s purpose, the voluntary nature of participation, complete anonymity, their right to withdraw at any time, and the academic use of aggregated findings. All data were anonymised at the point of collection through Microsoft Forms’ anonymous response setting, with no personally identifiable information collected. Distribution coordination by ministry HRM officers was logistical only, with no officer access to individual response-level data.

Note on Exploratory Factor Analysis (EFA) and Confirmatory Factor Analysis (CFA) Procedure: Both EFA and CFA were conducted on the same sample (*N* = 311) due to access constraints inherent in cross-sectional government ministry research. The EFA results are no longer presented as primary validity evidence. The CFA—grounded in the theoretically pre-specified factor structure of two validated, widely-used instruments (MWMS; MSQ-SF)—constitutes the sole confirmatory validity analysis. All 25 items loaded on their intended factors with standardised loadings of 0.62–0.81 and cross-loadings ≤ 0.30 in the CFA, confirming that the four-factor structure is theoretically coherent and not an artefact of sample-specific covariances. External cross-validation on an independent GCC government sample is recommended in future research.

### 3.5 Analytical strategy

The analytical framework distinguishes a single core analytical model from a set of supplementary analyses. The core analytical model is a first-stage moderated mediation model (PROCESS Model 7; Hayes, 2018) testing whether supervisory practices statistically mediate the motivation–satisfaction association, with gender and job level operating as boundary conditions on the X → M path. This single model evaluates H1–H3 (direct associations), H5 (indirect pathway), H6–H9 (boundary conditions), and H10 (Index of Moderated Mediation). Supplementary analyses are reported in service of, but distinct from, the core model: (a) H4—an exploratory bivariate test of the equity–satisfaction association using a single MSQ-SF item; (b) hierarchical regression (Table 1) testing moderation of the total X → Y effect, retained for descriptive completeness alongside the X → M decomposition produced by PROCESS Model 7; and (c) robustness checks [dummy-coded job level (Supplementary material S3); JS2-exclusion sensitivity re-analysis (Supplementary material S4); sensitivity power analyses for H7 and H9 (Supplementary material S6)], reported in summary form below and in full in the Supplementary material.

Data analysis covered four stages. First, descriptive statistics and Pearson correlations were conducted to assess inter-construct associations. Second, simultaneous-entry multiple regression was conducted for main effects (H1–H3). Third, PROCESS Macro Model 4 mediation with 5,000 bootstrap samples tested the indirect effects of supervisory practices (H5). Finally, hierarchical moderated regression and PROCESS Macro Model 7 moderated mediation (H6–H10) were conducted. Main effect, mediation, and moderation analyses were conducted using IBM SPSS Statistics 29 (IBM Corp, 2022a), with PROCESS Macro version 4.3 (Hayes, 2018) installed as a custom dialog box. CFA was conducted using IBM SPSS AMOS 29 (IBM Corp, 2022b). Continuous predictors were mean-centred prior to computing interaction terms to reduce non-essential multicollinearity (Aiken and West, 1991; Hayes, 2018). For H6–H9, the hierarchical regression (see Table 1) tested interaction effects in the full outcome equation (Y), providing an initial test of whether demographic variables moderated the overall motivation–satisfaction relationship. It should be noted that this hierarchical regression tests moderation of the total X → Y effect (motivation on satisfaction), and not the theoretically specified X → M path (motivation on supervisory practices) in isolation. PROCESS Model 7 (see Section 4.6) subsequently provides the inferentially appropriate decomposition, isolating the specific X → M path moderation consistent with the theoretical framework.

For moderation analyses involving Job Level, the three-level ordinal variable (Staff/Specialis*t* = 0; Supervisor/Manage*r* = 1; Director/Senior Manage*r* = 2) was treated as a quasi-continuous predictor following Hayes (2018, p. 249), who endorses this approach for ordered categorical moderators with three or more levels to enable estimation of the Index of Moderated Mediation. The equal-interval assumption is supported institutionally by Saudi government grading bands. Robustness of H8 and H9 to dummy-coded specification was verified; full dummy-coded estimates, simple slopes at each level, and partial f^2^ values are reported in Supplementary material S3. Simple slope probing in the main analysis was conducted at the Staff/Specialist (coded 0) and Director/Senior Manager (coded 2) levels as the theoretically meaningful contrast points (see Section 2.4). Throughout this manuscript, “moderated mediation” denotes the IMM test, and “conditional indirect effects” denotes the path-specific bootstrapped effects at specific moderator values (Hayes, 2018, Chapter 11).

All standard regression assumptions—normality of residuals, homoscedasticity, absence of multicollinearity (VIF < 3.0), and independence of errors—were verified and met conventional thresholds; full diagnostic statistics are reported in Supplementary material S12. Conditional slopes were computed at each actual coding level of the ordinal moderator (Job Level: 0 = Staff/Specialist; 1 = Supervisor/Manager; 2 = Director/Senior Manager) and the binary moderator (Gender: Male = 0; Female = 1), as the ordinal and dichotomous coding values themselves constitute the theoretically meaningful contrast points (Aiken and West, 1991).

## 4 Results

### 4.1 Descriptive statistics and correlations

Table 3 shows descriptive statistics by item and construct. Extrinsic motivation items recorded higher overall means (M range: 4.00–4.25) than intrinsic motivation items (M range: 3.72–4.14). The Job Satisfaction composite (three-item) had a mean of *M* = 3.46 (SD = 0.71); full item-level descriptive statistics for the three composite items are provided in Supplementary Appendix A. H4 [Exploratory]. The effort–reward balance item (the standalone equity indicator, excluded from the Job Satisfaction composite) recorded the lowest mean across all items (*M* = 3.22, SD = 0.89), indicating the participants typically perceived a degree of inequity in their effort–reward exchange. The item was significantly negatively correlated with the three-item Job Satisfaction composite [*r* = −0.34, 95% CI (−0.44, −0.24), *p* < 0.001]: employees reporting greater perceived inequity (lower effort–reward balance scores) reported lower job satisfaction, providing exploratory directional support for Adams (1965) Equity Theory prediction that perceived inequity in the effort–reward exchange is negatively associated with job satisfaction (Table 4).

**Table 3:** Descriptive statistics by item and construct (*N* = 311).

Item	M	SD	% Agree+	Rank
Intrinsic motivation (*α* = 0.84; *M* = 3.90)				
Sense of accomplishment from tasks	4.14	0.65	83.3%	1
Opportunities to learn new skills	3.92	0.71	77.8%	2
Work aligned with personal values	3.89	0.74	75.0%	3
Personal growth opportunities	3.72	0.82	66.7%	4
Extrinsic motivation (*α* = 0.87; *M* = 4.11)				
Supervisor recognition increases productivity	4.25	0.74	86.1%	1
Benefits and compensation maintain motivation	4.17	0.68	83.3%	2
Promotion opportunities increase effort	4.03	0.77	77.8%	3
Job security motivates performance	4.00	0.81	75.0%	4
Supervisory practices – mediator (*α* = 0.81; *M* = 3.58)				
Supervisor provides timely performance feedback	3.67	0.74	68.2%	1
Supervisor recognises employee contributions	3.61	0.77	65.9%	2
Job satisfaction (*α* = 0.83; *M* = 3.46)				
Overall satisfied with current job	3.53	0.79	62.1%	2
Balance between effort and reward†	3.22	0.89	48.6%	4

† = Effort–reward balance item; operationalised as a standalone single-item equity indicator for the H4 test (Adams’ Equity Theory), excluded from the three-item Job Satisfaction composite. It records the lowest mean across all items (*M* = 3.22), indicating that on average participants perceived the greatest degree of inequity on this item relative to all other items, consistent with H4. Representative items are shown; the Job Satisfaction section displays one of the three composite items (the other two composite items—including satisfaction with career progression opportunities—are omitted for space); the full item list is listed in Supplementary Appendix A. Ranks reflect position within the full construct item set; not all ranks are displayed for abbreviated constructs. The effort–reward balance item (Rank 4) ranks 4th out of all four MSQ-SF items originally included in the Job Satisfaction section (including the three composite items plus this standalone indicator); its rank among composite items only is not applicable as it was excluded from the composite prior to analysis.

**Table 4:** Inter-construct Pearson correlation matrix (*N* = 311).

Construct	M	SD	1	2	3	4
1. Intrinsic motivation	3.90	0.67	(0.73)			
2. Extrinsic motivation	4.11	0.68	0.33***	(0.72)		
3. Supervisory practices	3.58	0.66	0.48***	0.57***	(0.71)	
4. Job satisfaction	3.46	0.71	0.53***	0.64***	0.61***	(0.71)

***p < 0.001 (two-tailed). All correlations are statistically significant. Cronbach’s α values: Intrinsic Motivatio*n* = 0.84; Extrinsic Motivatio*n* = 0.87; Supervisory Practices = 0.81; Job Satisfaction (3-item composite) = 0.83. Inter-construct correlations are Pearson r; all *p* < 0.001. Diagonal entries in parentheses report the square root of the Average Variance Extracted (√AVE) for each construct: IM (√AVE = 0.73); EM (√AVE = 0.72); Supervisory Practices (√AVE = 0.71); Job Satisfaction (√AVE = 0.71). Fornell–Larcker discriminant validity is supported when √AVE exceeds all off-diagonal inter-construct correlations in the same row/column: all four √AVE values satisfy this criterion, confirming discriminant validity for all construct pairs. Note that the Supervisory Practices–Job Satisfaction pair is the most proximal (√AVE = 0.71 vs. *r* = 0.61; difference = 0.10), as expected given shared MSQ-SF item origin; all other pairs show larger margins. 95% confidence intervals (Fisher’s Z transformation, *N* = 311, SE_Z = 0.057): IM–EM *r* = 0.33 [0.23, 0.43]; IM–Sup *r* = 0.48 [0.39, 0.56]; IM–JS *r* = 0.53 [0.45, 0.61]; EM–Sup *r* = 0.57 [0.49,0.64]; EM–JS *r* = 0.64 [0.57, 0.70]; Sup–JS *r* = 0.61 [0.54, 0.68].

### 4.2 Main effects: multiple regression (H1–H3)

Table 5 shows simultaneous-entry regression results. The main effects model explains 52.4% of variance in job satisfaction [R^2^ = 0.524, *F*(2,308) = 169.29, *p* < 0.001]. Extrinsic motivation (*β* = 0.52, *t* = 12.53, *p* < 0.001) more strongly predicted job satisfaction than intrinsic motivation (*β* = 0.36, *t* = 8.59, *p* < 0.001), supporting H1-H3. To formally test H3, the difference between standardised regression coefficients was evaluated using the variance–covariance matrix of the regression estimates. With SE_EM = 0.043, SE_IM = 0.044, and the covariance between the two estimates accounted for, SE_diff = 0.057, yielding z = (0.52–0.36)/0.057 = 2.81, *p* = 0.005 (two-tailed; Clogg et al., 1995; Cohen et al., 2003), providing inferential support for the hypothesis that extrinsic motivation is a significantly stronger predictor than intrinsic motivation.

**Table 5:** Multiple regression results: main effects on job satisfaction (*N* = 311).

Predictor	*B*	SE B	*β*	*t*	*P*
Constant	−0.24	0.23		−1.03	0.320
Intrinsic motivation	0.38	0.044	0.36***	8.59	< 0.001
Extrinsic motivation	0.54	0.043	0.52***	12.53	< 0.001

R^2^ = 0.524; F(2,308) = 169.29, *p* < 0.001. Dependent variable: Job satisfaction. Predictors entered simultaneously (simultaneous-entry method); no sequential ordering is implied. ***p < 0.001.

### 4.3 Equity theory test (H4) and mediation analysis (H5)

Consistent with H4 [Exploratory], the effort–reward balance item (treated as a standalone indicator, excluded from the Job Satisfaction composite - see Section 3.3) was significantly negatively correlated with the three-item Job Satisfaction composite [*r* = −0.34, 95% CI (−0.44, −0.24), *p* < 0.001], providing correlational support for Adams (1965) Equity Theory and indicating that perceived inequity in the effort–reward exchange is independently associated with lower job satisfaction.

H4 was assessed as an exploratory bivariate test rather than as a modelled pathway, reflecting the single-item operationalisation of the effort–reward balance indicator. Result is reported in-text rather than in a separate table. The single-item measure means reliability is unknown and the result must be interpreted with caution; replication with a validated multi-item equity measure (e.g., Siegrist’s Effort–Reward Imbalance scale) is required before substantive theoretical conclusions can be drawn (see Section 5.7).

Table 6 presents PROCESS Macro Model 4 mediation results. Partial mediation through supervisory practices was confirmed for both motivational pathways. The indirect effect of extrinsic motivation was significant [*b* = 0.21, standard error (SE) = 0.03, 95% BC CI (0.14, 0.30)], as was the indirect effect of intrinsic motivation [*b* = 0.11, SE = 0.03, 95% BC CI (0.05, 0.17)]. Both direct effects remained significant after controlling for the mediator, confirming partial mediation and supporting H5. It should be noted that the arithmetic sums of direct and indirect effects in Table 6 (EM: 0.33 + 0.21 = 0.54; IM: 0.27 + 0.11 = 0.38) numerically equal the Table 5 total-effect coefficients (*B* = 0.54; *B* = 0.38). This numerical agreement shows that the bias-corrected bootstrapped indirect effects, when summed with the partial direct effects, recover the total-effect magnitude reported in Table 5. Both mediation pathways confirm partial mediation: the direct effects remain significant after the mediator is controlled, and the bias-corrected confidence intervals exclude zero, supporting H5. The effect decomposition confirms that extrinsic motivation exceeds intrinsic motivation in total, direct, and indirect effects simultaneously (EM: B_total = 0.54, B_direc*t* = 0.33, B_indirec*t* = 0.21; IM: B_total = 0.38, B_direc*t* = 0.27, B_indirec*t* = 0.11), rendering H3 and H5 fully consistent: EM dominates all three quantities, and no suppressor effects or compensatory patterns are present in the data.

**Table 6:** Mediation results: PROCESS macro model 4 (*N* = 311; 5,000 bootstrap samples).

Path	b Direct	b Indirect	SE	95% BC CI	Mediation
Extrinsic → Supervision → Satisfaction	0.33***	0.21	0.03	[0.14, 0.30]	Partial
Intrinsic → Supervision → Satisfaction	0.27***	0.11	0.03	[0.05, 0.17]	Partial

BC CI = Bias-corrected 95% Confidence Interval. Neither CI includes zero, confirming significance of indirect effects (bootstrap CIs do not yield *p*-values; Hayes, 2018). ***p < 0.001 applies to direct effects only. *b* = unstandardised regression coefficient. Direct effects in this model reflect the controlled (partial) effect of each motivational predictor on Job Satisfaction after accounting for the supervisory mediator; when summed with the indirect effects, they recover the total-effect coefficients in Table 5 (EM: 0.33 + 0.21 = 0.54; IM: 0.27 + 0.11 = 0.38). The indirect effects and CIs are the inferentially relevant quantities for H5.

### 4.4 Moderation analysis (H6–H9)

Table 1 presents hierarchical regression results. As noted in Section 3.5, the interaction terms in this table test moderation of the total motivation–satisfaction (X → Y) relationship; the theoretically specified X → M path moderation is formally tested in PROCESS Model 7 (Section 4.6). The demographic-only Model 1 (R^2^ = 0.047) confirms that gender and job level account for a small but non-trivial share of variance in job satisfaction independently of motivational processes, consistent with the expectation that structural position influences workplace outcomes in hierarchical organisations. Adding motivational predictors in Model 2 raised the explained variance substantially (R^2^ = 0.524), and addition of interaction terms in Model 3 produced a significant incremental R^2^ change (ΔR^2^ = 0.090, *F*(4,302) = 17.80, *p* < 0.001), raising total explained variance to 61.4% in the final moderated model. Two interactions were significant: Gender × Intrinsic Motivation (βIN*T* = 0.18, *p* = 0.003), supporting H6; and Job Level × Extrinsic Motivation (βIN*T* = −0.14, *p* = 0.021), supporting H8. H7 and H9 were not supported. Throughout Sections 4.4–5.4, βINT denotes the standardised regression coefficient for the interaction (product) term in the hierarchical moderated regression model.

The non-significant Gender × Extrinsic Motivation interaction (βIN*T* = 0.07, *p* = 0.214) indicates that H7 is not supported; notably, the positive direction of this coefficient (Male = 0, Female = 1) indicates that, if anything, female employees showed marginally stronger extrinsic motivational effects than male employees—the opposite direction to H7’s prediction—though the difference was negligible and far from significant. Similarly, the non-significant Job Level × Intrinsic Motivation interaction (βIN*T* = 0.06, *p* = 0.287) suggests that H9 is not supported; the positive direction of this coefficient is broadly consistent with the prediction of increasing intrinsic salience at higher job levels, but the effect was too small to reach significance, suggesting that intrinsic motivation maintains relatively stable predictive strength across hierarchical levels in this sample. Sensitivity power analyses (G*Power 3.1; Faul et al., 2007) confirmed adequate power to detect small-to-medium interactions (minimum detectable f^2^ = 0.037; full computations in Supplementary material S6), supporting interpretation of the H7 and H9 null findings as true absence of moderation rather than Type II error. Theoretical interpretation is developed in Section 5.

### 4.5 Simple slopes analysis

**Figure 2:**
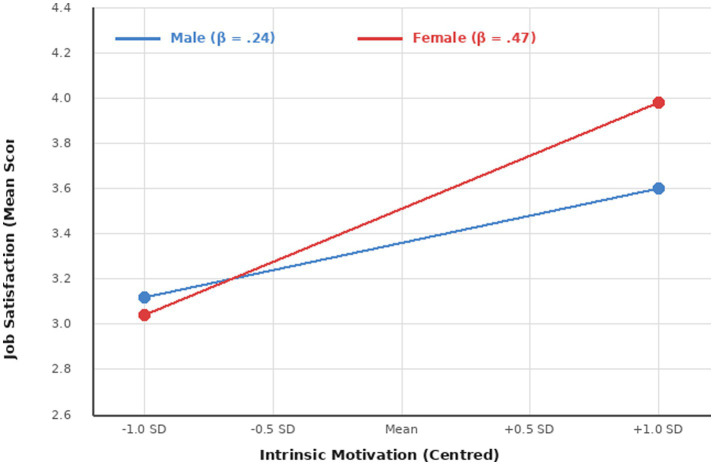
Simple slopes: gender moderation of intrinsic motivation on job satisfaction. Simple slopes plotted separately for male employees (coded 0) and Female employees (coded 1) across the observed range of Intrinsic Motivation scores. Slopes derived from hierarchical regression Model 3 (Table 1). Male slope: *β* = 0.24, SE = 0.06, t(302) = 4.00, *p* < 0.001, 95% CI [0.12, 0.36]. Female slope: *β* = 0.47, SE = 0.08, t(302) = 5.88, *p* < 0.001, 95% CI [0.31, 0.63]. The female slope is significantly steeper (z = 2.30, *p* = 0.021), indicating that intrinsic motivation is a stronger predictor of job satisfaction for female employees. Shaded bands represent ±1 SE around each slope.

Simple slopes analysis (Aiken and West, 1991) was conducted to probe the two statistically significant interactions (H6 and H8). For the Gender × Intrinsic Motivation interaction (H6 supported), intrinsic motivation more strongly predicted job satisfaction for female employees [*β* = 0.47, SE = 0.08, t(302) = 5.88, *p* < 0.001] than for their male counterparts [*β* = 0.24, SE = 0.06, t(302) = 4.00, *p* < 0.001]. The difference in slopes was significant (*z* = 2.30, *p* = 0.021). These conditional effects are presented in Table 7 and visualised in Figure 2.

**Table 7:** Simple slopes: intrinsic motivation predicting job satisfaction at each level of gender.

Gender	Β	SE	t(302)	P	95% CI
Male (coded 0)	0.24	0.06	4.00	< 0.001	[0.12, 0.36]
Female (coded 1)	0.47	0.08	5.88	< 0.001	[0.31, 0.63]

Simple slopes derived from the hierarchical regression Model 3. Both slopes are statistically significant, with the female slope significantly steeper (*z* = 2.30, *p* = 0.021). 95% CIs computed as *β* ± 1.967 × SE (t critical, df = 302, α = 0.05, two-tailed).

**Figure 3:**
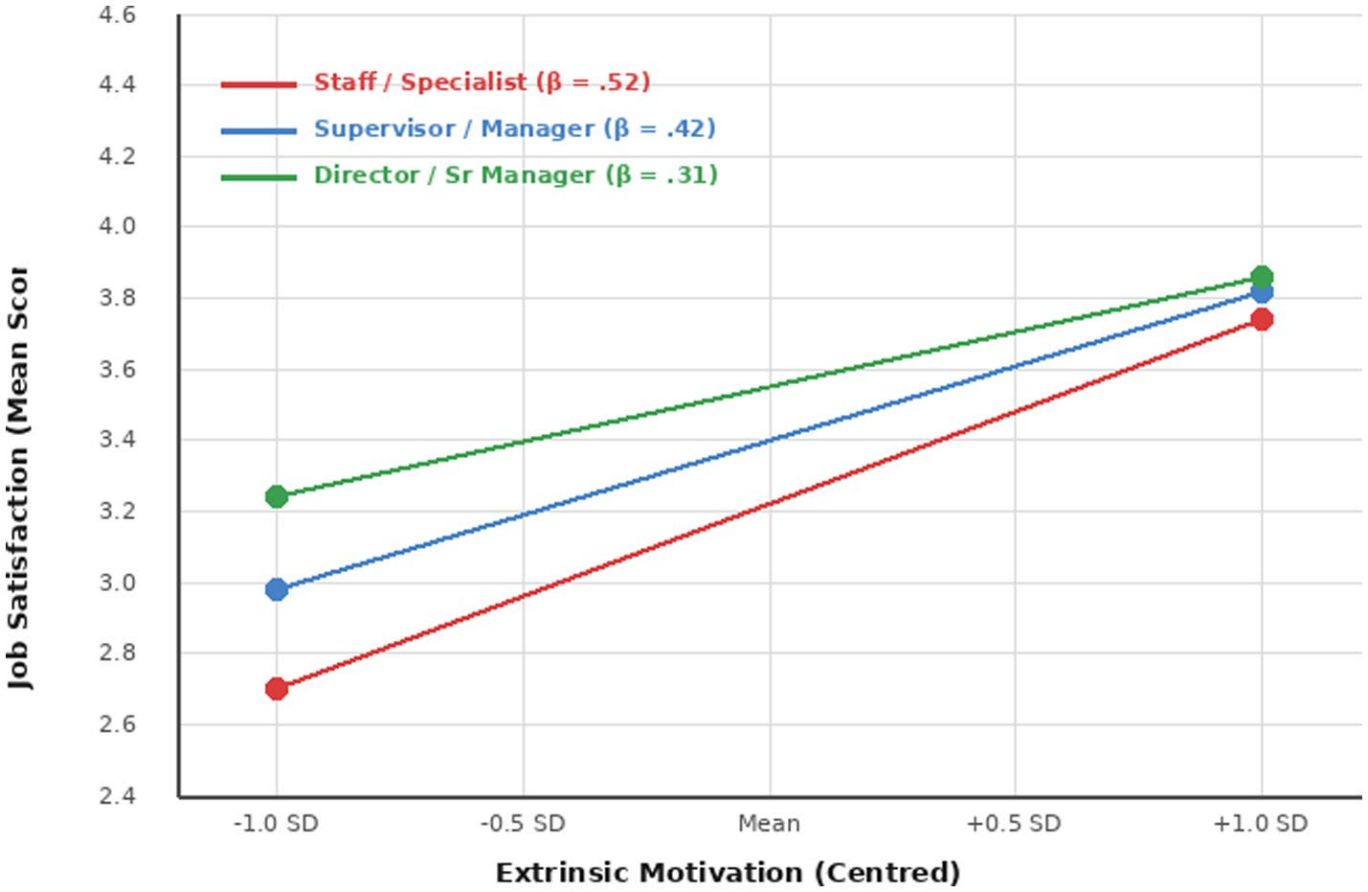
Simple slopes: job level moderation of extrinsic motivation on job satisfaction. Simple slopes plotted separately for staff/specialist (coded 0), supervisor/manager (coded 1), and Director/Senior manager (coded 2) across the observed range of extrinsic motivation scores. Slopes derived from hierarchical regression Model 3 (Table 1). Staff slope: *β* = 0.52, SE = 0.09, t(302) = 5.78, *p* < 0.001, 95% CI [0.34, 0.70]. Supervisor slope: *β* = 0.42, SE = 0.07, t(302) = 6.00, *p* < 0.001. Director slope: *β* = 0.31, SE = 0.10, t(302) = 3.10, *p* = 0.002, 95% CI [0.11, 0.51]. The steeper Staff slope indicates stronger extrinsic motivation effects at lower hierarchical levels, consistent with the need-hierarchy prediction that lower-level needs carry greater motivational salience at lower organisational levels. The formal moderation test (H8) contrasts Staff vs. Director levels as the theoretically specified extremes; see Section 3.5.

For the Job Level × Extrinsic Motivation interaction (H8 supported), extrinsic motivation was a significantly stronger predictor for Staff-level employees [Job Level coded 0: *β* = 0.52, SE = 0.09, t(302) = 5.78, *p* < 0.001] than for Director-level employees [Job Level coded 2: *β* = 0.31, SE = 0.10, t(302) = 3.10, *p* = 0.002]. These conditional effects are shown in Table 8 and visualised in Figure 3.

**Table 8:** Moderated mediation results: PROCESS macro model 7 (*N* = 311; 5,000 Bootstrap Samples).

Conditional indirect effect	Moderator level	b Indirect	SE	95% BC CI	Sig.
Intrinsic → Supervision → Satisfaction (Moderated by Gender)	Male (coded 0)	0.06	0.02	[0.02, 0.11]	Yes ✓
	Female (coded 1)	0.14	0.03	[0.08, 0.21]	Yes ✓
*Index of moderated mediation (IMM)*		0.08	0.03	[0.02, 0.15]	Yes ✓
Extrinsic → Supervision → Satisfaction (Moderated by Job Level)	Staff/Specialist (coded 0)	0.19	0.04	[0.12, 0.27]	Yes ✓
	Director/Senior manager (coded 2)	0.09	0.04	[0.03, 0.16]	Yes ✓
*Index of moderated mediation (IMM)*		−0.05	0.04	[−0.09, −0.02]	Yes ✓

BC CI = Bias-Corrected 95% Confidence Interval. No CI includes zero, confirming significance of all moderated mediation effects. *b* = unstandardised regression coefficient. SE values report the standard deviation of the bootstrap sampling distribution; inference is based on the bias-corrected CI, which accounts for asymmetry and bias in the bootstrap distribution and should not be used to construct normal-approximation confidence intervals for indirect effects (Hayes, 2018, p. 97). Job Level coding: 0 = Staff/Specialist, 1 = Supervisor/Manager, 2 = Director/Senior Manager (consistent with Table 2 coding scheme throughout).

### 4.6 Moderated mediation (H10)

Table 8 presents PROCESS Macro Model 7 results. Note that the slopes reported in Table 9 (from the total-effect hierarchical regression without a mediator) cannot be directly compared to the conditional indirect effects reported here: Table 8 decomposes the total effect into a direct path and an indirect path through the supervisory mediator, so the figures reflect different analytical quantities and the apparent numerical difference between the two tables is not unexpected. Significant moderated mediation (IMM test) was confirmed for two pathways, with conditional indirect effects varying meaningfully across moderator levels. The conditional indirect effect of intrinsic motivation through supervisory practices was significantly larger for female employees [*b* = 0.14, SE = 0.03, 95% BC CI (0.08, 0.21)] than for males [*b* = 0.06, SE = 0.02, 95% BC CI (0.02, 0.11)]. The IMM was significant [IMM = 0.08, 95% BC CI (0.02, 0.15)], confirming first-stage moderated mediation (IMM = 0.08). The conditional indirect effects of the extrinsic motivation pathway were moderated by job level and was stronger for Staff-level employees [*b* = 0.19, SE = 0.04, 95% BC CI (0.12, 0.27)] than for Director-level employees [*b* = 0.09, SE = 0.04, 95% BC CI (0.03, 0.16)], with IMM = −0.05, 95% BC CI [−0.09, −0.02]. The conditional indirect effect at the intermediate Supervisor/Manager level (*b* = 0.14, SE = 0.03) fell between these extremes, consistent with the linear hierarchical gradient; it is omitted from Table 8 for simplicity as the formal moderation test (H8) is specified at the theoretically meaningful extreme levels (see Section 3.5). H10 is supported.

**Table 9:** Simple slopes: extrinsic motivation predicting job satisfaction at each level of job level.

Job level	*β*	se	t(302)	*p*	95% ci
Staff/Specialist (coded 0)	0.52	0.09	5.78	< 0.001	[0.34, 0.70]
Supervisor/Manager (coded 1)	0.42	0.07	6.00	< 0.001	[0.28, 0.56]
Director/Senior manager (coded 2)	0.31	0.10	3.10	0.002	[0.11, 0.51]

Simple slopes derived from the hierarchical regression Model 3 using Job Level coding: 0 = Staff/Specialist, 1 = Supervisor/Manager, 2 = Director/Senior Manager. The Supervisor/Manager level is reported here for completeness of the hierarchical gradient; however, the formal moderation test (H8) is specified at the theoretically meaningful extremes of Staff/Specialist (coded 0) and Director/Senior Manager (coded 2), consistent with the analytical strategy detailed in Section 3.5. 95% CIs computed as *β* ± 1.967 × SE (t critical, df = 302, *α* = 0.05, two-tailed).

### 4.7 Hypothesis testing summary

Table 10 presents a consolidated summary of the hypothesis testing results. The framework distinguishes nine confirmatory hypotheses (H1–H3, H5–H10) and one exploratory hypothesis (H4). Among the confirmatory hypotheses, seven were supported and two (H7, H9) were not. The exploratory test of equity (H4) yielded a directionally consistent association, but should be replicated with a validated multi-item instrument before substantive theoretical conclusions are drawn.

**Table 10:** Hypothesis testing summary.

H	Hypothesis	Key result	Status
H1	Extrinsic motivation significantly predicts job satisfaction	*β* = 0.52, *p* < 0.001	✓ Supported
H2	Intrinsic motivation significantly predicts job satisfaction	*β* = 0.36, *p* < 0.001	✓ Supported
H3	Extrinsic motivation is a significantly stronger predictor than intrinsic motivation (formal z-test of coefficient difference: z = 2.81, *p* = 0.005)	0.52 > 0.36	✓ Supported
H4 [Exploratory]	Perceived effort–reward inequity negatively associated with job satisfaction	*r* = −0.34, 95% CI [−0.44, −0.24], *p* < 0.001	✓ Directionally consistent (exploratory)
H5	Supervisory practices partially mediate motivation–satisfaction	Partial mediation confirmed	✓ Supported
H6	Gender moderates intrinsic motivation path (female stronger)	βIN*T* = 0.18, *p* = 0.003	✓ Supported
H7	Gender moderates extrinsic motivation path	βIN*T* = 0.07, ns	× Not supported
H8	Job level moderates extrinsic path (lower level stronger)	βIN*T* = −0.14, *p* = 0.021	✓ Supported
H9	Job level moderates intrinsic motivation path	βIN*T* = 0.06, ns	× Not supported
H10	Moderated mediation through supervisory practices	IMM significant (both pathways)	✓ Supported

✓Supported; ×Not supported; ns, non-significant; IMM, index of moderated mediation. Key results report standardised coefficients (β) and p-values as derived from the analyses in Tables 5–8. Significance thresholds: *p* < 0.001 (H1, H2, H4); *p* < 0.01 (H3: *p* = 0.005; H6: *p* = 0.003); *p* < 0.05 (H8: *p* = 0.021). H4 is reported here for completeness but is methodologically distinct from the nine confirmatory hypotheses (H1–H3, H5–H10): it relies on a single-item operationalisation of the equity construct and is therefore treated as an exploratory test outside the core analytical model (see Sections 3.5 and 5.7). Substantive theoretical conclusions about Adams (1965) equity prediction should not be drawn from this row alone and require multi-item replication.

## 5 Discussion

A note on interpretive language. All relationships reported in this section—including direct effects, indirect effects through the supervisory pathway, and conditional indirect effects—are cross-sectional statistical associations. Where the manuscript uses terms such as “pathway,” “mechanism,” or “mediation,” these are intended in their statistical-decompositional sense (a₁·b in Hayes’s PROCESS notation) and should not be read as claims of directional causality. Causal inference would require longitudinal panel data with appropriate identification strategies, which constitutes a primary direction for future research (see Section 5.7).

### 5.1 Challenging Herzberg in a collectivist context (H1–H3)

Extrinsic motivation showed a larger standardised association with job satisfaction (*β* = 0.52) than intrinsic motivation (*β* = 0.36; z = 2.81, *p* = 0.005), a pattern inconsistent with Herzberg (1966) asymmetric prediction that only motivators are associated with genuine satisfaction. This pattern is consistent with evidence from other GCC studies finding that extrinsic conditions are substantial predictors of employee attitudes in relational exchange contexts (e.g., AlMarzooqi et al., 2025) and with Manzoor et al. (2021) finding that both intrinsic and extrinsic motivational factors jointly predict work outcomes in Pakistani organisational contexts, extending these findings to the Saudi central government context.

The proposed associational pattern is theoretically identifiable. Standardised grading systems in Saudi public bodies structurally constrain the performance–reward link, leaving supervisory recognition as the primary available valence-linked outcome (Vroom, 1964); supervisor recognition was correspondingly the highest-rated extrinsic item (*M* = 4.25). SDT’s relatedness prediction (Deci and Ryan, 2000) anticipates elevated motivational weight for relational supervisory inputs in collectivist, high power-distance environments—a prediction consistent with the cross-sectional patterns observed here.

Effect sizes are contextually meaningful. The standardised regression coefficients obtained here (*β* = 0.52 for extrinsic motivation; *β* = 0.36 for intrinsic motivation) are notably large relative to the benchmarks Cohen (1988) proposes for behavioural science. The overall model f^2^ = R^2^/(1 − R^2^) = 0.524/0.476 = 1.10 exceeds Cohen’s (1988) large-effect threshold of f^2^ = 0.35 for multiple regression, indicating that the model explains a substantial proportion of variance in job satisfaction. It should be noted, however, that f^2^ values of this magnitude are uncommon in field survey data and should thus be interpreted with due caution.

Although the multi-procedure CMB diagnostics (see Section 3.3) indicate no critical method inflation, the possibility of residual common method variance contributing to the elevated explained variance cannot be entirely excluded. In particular, the shared MSQ-SF origin of the Supervisory Practices mediator and the Job Satisfaction outcome (inter-construct *r* = 0.61) may have contributed to correlation elevation between these two constructs beyond the level their theoretical proximity alone might generate. This concern is mitigated by the multi-procedure discriminant validity evidence reported in Section 3.3, including the Fornell–Larcker criterion, non-overlapping item content evaluation, and the full collinearity VIF assessment. HTMT ratios (range: 0.44–0.77) were computed for all construct pairs using item-level analysis in R (Revelle, 2023) and all fell below the conservative 0.85 threshold (Henseler et al., 2015), providing additional discriminant validity support beyond the Fornell–Larcker criterion (see Section 5.7).

In comparative terms, these coefficients exceed the motivation–satisfaction *β* values reported in analogous GCC public-sector studies (AlMarzooqi et al., 2025; Aljumah, 2023), suggesting that the particular institutional configuration of Vision 2030’s reform context may amplify the motivational responsiveness of job satisfaction beyond that typically observed in Western organisational samples—a pattern consistent with Hofstede’s (2001) prediction that interpersonal dynamics carry disproportionate motivational weight in high power-distance institutions. The present findings are inconsistent with the universality of Herzberg’s hygiene–motivator hierarchy, showing that extrinsic conditions carry motivational weight not predicted by his original theory in these institutional and cultural conditions.

### 5.2 Supervisory practices as active mediating pathway (H5)

H5 is supported. Supervisory communication and recognition partially mediated both motivational pathways in associational terms. This indicates that supervisory practices statistically operate as an active mediating pathway rather than functioning as a passive contextual background variable; the term “mediating pathway” is used here in its statistical-decompositional sense (a₁·b in Hayes’s PROCESS notation; see Section 5 opening note) and does not imply directional causality given the cross-sectional design. Two aspects of this mediation warrant theoretical attention. First, the indirect effect was stronger for extrinsic motivation (*b* = 0.21) than intrinsic motivation (*b* = 0.11), an asymmetry consistent with Blau’s (1964) social exchange prediction: in high power-distance institutions, the supervisor–employee relational exchange is more tightly associated with extrinsic motivational inputs. Second, partial rather than full mediation indicates that direct motivational effects persist independently of supervisory practices. This finding—that multiple pathways from motivation to satisfaction are thus operating simultaneously—extends Cho and Perry’s (2012) observation that supervisory support functions as a boundary condition rather than an exclusive mediating channel in public-sector motivation research.

The interpretation of these mediation findings should be tempered by an important methodological caveat. Because the supervisory practices mediator and the job satisfaction outcome were operationalised through items adapted from the same source instrument (MSQ-SF), the observed indirect effects may be partially attributable to shared instrument variance that statistical discriminant-validity diagnostics (HTMT, Fornell–Larcker) cannot fully resolve at the conceptual level. The indirect-effect magnitudes reported here should therefore be read as upper-bound associational estimates, and the substantive conclusion that supervisory practices function as an active mediating pathway requires confirmation in studies that operationalise the supervisory mediator through a purpose-designed instrument (e.g., LMX-7; Graen and Uhl-Bien, 1995) measured independently of the satisfaction outcome.

### 5.3 Gender moderation: vision 2030 workforce evidence (H6, H7)

Support for H6 constitutes this study’s most novel empirical contribution: intrinsic motivation is a significantly stronger predictor for female employees (*β* = 0.47) than for males (*β* = 0.24).

The theoretical interpretation of this finding follows social identity theory (Tajfel and Turner, 1986). Female employees entering newly accessible government roles under Vision 2030 derive stronger satisfaction from task meaningfulness and value alignment. Work participation itself carries identity-affirming significance in a context of structural role transition. The motivational weight of intrinsic inputs is therefore elevated beyond its instrumental value.

H7 is not supported (βIN*T* = 0.07, *p* = 0.214). Notably, the observed positive direction of this coefficient (Male = 0, Female = 1) indicates that, if anything, female employees had marginally stronger extrinsic motivational effects than males—inconsistent with H7’s predicted direction—though the difference was negligible and far from significant. This null finding does not straightforwardly falsify Eagly’s (1987) social role theory. It is important to note, however, that the cross-sectional design captures a single time-point and cannot distinguish a reform-induced motivational shift from a pre-existing demographic characteristic of this workforce; longitudinal comparison data covering pre- and post-reform periods would be required to confirm a causal reform effect. With that caveat, one theoretically consistent interpretation is that gender-based extrinsic motivational differentiation diminishes as structural role access becomes more equitable: under the Vision 2030 reforms, female employees now occupy roles with equivalent salary grades, job security entitlements, and promotion pathways traditionally dominated by their male counterparts, which may have eroded the gender gap in extrinsic motivational salience that social role theory would predict in more differentiated role environments. But the practical implication is precise: while extrinsic recognition strategies require no gender differentiation in the current workforce, intrinsic enrichment interventions do.

### 5.4 Job level moderation: hierarchical need satisfaction (H8, H9)

H8 is supported. Extrinsic motivation produced a monotonic decrease in effect size across the hierarchical gradient: Staff (*β* = 0.52), Supervisor/Manager (*β* = 0.42), Director (*β* = 0.31). This pattern concurs with need-hierarchy predictions of declining extrinsic salience as lower-order needs are more adequately met at higher job levels.

H9 is not supported (β_int_ = 0.06, *p* = 0.287). Intrinsic motivation maintained stable predictive strength across all three job levels, contrasting with the need-hierarchy prediction of increasing intrinsic salience and aligning instead with ERG predictions: when growth-need satisfaction is structurally denied (as under Saudi public grading systems with their promotion ceilings and role rigidity), intrinsic motivation maintains uniform salience across hierarchical positions. The H9 non-support is therefore not a failure of need-hierarchy logic but evidence that the institutional architecture of the Saudi public sector suppresses the hierarchical need gradient that classical hierarchy theories presuppose.

The divergence between H8 support and H9 non-support reveals a theoretically productive asymmetry: extrinsic needs exhibit the hierarchical gradient predicted by need theories; intrinsic needs exhibit the uniform salience predicted by frustration-regression dynamics in structurally constrained environments.

### 5.5 Moderated mediation (H10)

H10 is supported for both boundary conditions. The supervisory indirect effect was 0.08 units larger per unit increase in intrinsic motivation for females relative to males [IMM = 0.08, 95% BC CI (0.02, 0.15)]. The supervisory indirect effect decreased by 0.05 units per unit increase in job level coding [IMM = −0.05, 95% BC CI (−0.09, −0.02)]. Both IMM coefficients are statistically significant (BC CIs do not include zero), confirming first-stage moderated mediation across both moderators.

These IMM coefficients have potential implications for HRM practice, although the underlying effect sizes are modest by Cohen’s (1988) conventions and warrant cautious interpretation. The conditional indirect effect of intrinsic motivation through supervisory practices was statistically larger for female (*b* = 0.14) than male employees (*b* = 0.06), and the conditional indirect effect of extrinsic motivation was statistically larger at the Staff/Specialist level (*b* = 0.19) than at the Director level (*b* = 0.09). Whether these statistical differences correspond to practically meaningful HRM-relevant differentials should be examined through pilot intervention designs before any general policy inference is drawn.

Ministry HRM directors face two targeted intervention priorities. First, to strengthen the conditional indirect association linking intrinsic motivation to satisfaction (H6, H10), supervisors managing female staff should be trained in meaningful task assignment, skill-based recognition, and career development dialog. Second, front-line supervisors managing entry-level staff require training in equitable reward communication, transparent promotion criteria, and material recognition practices to support the conditional indirect association linking extrinsic motivation to satisfaction (H8, H10). Both recommendations are applicable under Vision 2030’s Human Capability Development Program (CEDA, 2021). Given the cross-sectional design, ministries should pilot these differentiated supervisory strategies in a representative subset of units (e.g., two to three departments per ministry) with pre-post satisfaction measurement before ministry-wide rollout.

### 5.6 Theoretical synthesis: a preliminary constrained need activation proposition

This study addressed one central question: do Herzberg’s, Adams’, and SDT’s predictions hold under the simultaneous pressures of cultural collectivism, accelerating gender workforce integration, and hierarchical role differentiation in Saudi public bodies under the Vision 2030 reform initiative?

The answer is structured and theoretically precise. Herzberg’s hygiene–motivator boundary does not hold: extrinsic factors carry motivational weight that meets and exceeds that of intrinsic factors (*z* = 2.81, *p* = 0.005), and supervisory recognition—a hygiene factor in Herzberg’s classification—operates as an active mediating pathway in associational terms (H1–H3, H5). Adams (1965) equity prediction is directionally supported (exploratory) as an independent satisfaction determinant through a clean bivariate test (effort–reward balance item correlated with the part-excluded three-item composite, *r* = −0.34; H4). SDT’s relatedness prediction is supported through the supervisory mediation pathway, and extended by the demographically contingent IMM result: supervisory practices are associated with stronger satisfaction returns for female employees and entry-level staff (H5, H10).

The non-supported hypotheses sharpen the framework. H7 non-support indicates extrinsic motivational dynamics are gender-convergent under Vision 2030’s structural reforms, partially challenging the social role theory prediction of male-dominant extrinsic salience—a divergence most plausibly attributable to Vision 2030’s structural cross-gender equalisation of salary grades and promotion routes (see Section 5.3). H9 non-support indicates that intrinsic motivation functions as a uniform predictor irrespective of hierarchical position, inconsistent with the need-hierarchy prediction of increasing intrinsic salience along the promotion ladder. As Section 5.4 set out, this outcome is more convincingly explained by Alderfer’s (1969) ERG frustration-regression dynamics under structurally constrained Saudi public grading systems. Together, these seven supported and two non-supported confirmatory hypotheses, plus one exploratory test (H4) yielding a directionally consistent association, comprise a coherent, institutionally grounded motivation–satisfaction framework for GCC public-sector HRM research.

Drawing these findings together into a tentative interpretive lens, we describe this pattern as the Constrained Need Activation (CNA) idea—offered here as a preliminary, context-specific conceptual proposition rather than a fully established framework, and one that requires future replication across independent samples and settings before broader theoretical claims can be made. As a preliminary proposition: in collectivist, high power-distance institutional environments where structural reward channels are constrained by standardised grading systems, supervisory recognition appears to emerge as the primary mediating pathway statistically associating motivational inputs with satisfaction outcomes in this single cross-sectional sample, with activation strength systematically conditioned by demographic boundary positions (gender and hierarchical level). The CNA proposition integrates three observations arising from the present findings, each requiring future replication.

First, a boundary-collapse proposition: Herzberg’s (1966) hygiene–motivator distinction does not appear to hold in this environment, as structural constraint of formal reward channels elevates the motivational salience of relational supervisory inputs that Herzberg classified as merely contextual.

Second, a demographic-activation proposition: the supervisory mediation pathway does not operate uniformly but is systematically conditioned by demographic position, extending Self-Determination Theory’s (Deci and Ryan, 2000) relatedness prediction by specifying when relational mediation is most consequential—for female employees navigating structural role transitions and for entry-level employees with unmet material needs.

Third, a hierarchical-asymmetry proposition: extrinsic salience declines monotonically across job levels (consistent with Maslow, 1943), while intrinsic salience remains uniform under structurally constrained growth-need satisfaction (consistent with ERG dynamics; Alderfer, 1969). CNA is therefore offered as a preliminary recombination of pre-existing theoretical accounts (SDT, Hofstede, ERG) tentatively reorganised around the structural conditions of the Saudi public sector observed in this single cross-sectional sample. The exploratory contribution is the recombination itself, not the constituent propositions, and it requires replication before any theoretical claim can be made.

For conceptual precision, the present analysis rests on three core theoretical pillars: Herzberg (1966) Two-Factor Theory (the focal account whose hygiene–motivator boundary is empirically tested), Self-Determination Theory’s (Deci and Ryan, 2000) relatedness prediction (the theoretical basis for supervisory mediation), and Social Identity Theory (Tajfel and Turner, 1986) (the basis for the gender-moderation hypothesis). The remaining frameworks invoked above—Adams (1965) Equity Theory, Maslow (1943) need hierarchy, Alderfer (1969) ERG dynamics, Blau (1964) social exchange theory, Vroom (1964) expectancy theory, and Hofstede (2001) cultural-dimensions logic—are drawn upon as supporting interpretive lenses for specific findings rather than as central theoretical scaffolding. This deliberate prioritisation responds to the need for theoretical economy: the CNA proposition is most parsimoniously read as a context-specific extension of the Herzberg–SDT–Social Identity nexus under structural reward constraint, with the supporting frameworks invoked only where the data prompt their explanatory relevance.

### 5.7 Limitations of the study

Notwithstanding the contributions outlined above, several limitations warrant acknowledgement, presented in order of inferential importance. First, the cross-sectional design precludes causal inference; all reported associations should be interpreted as cross-sectional co-variations rather than evidence of directional influence. Second, the model does not include controls for theoretically relevant variables that may jointly influence motivation and satisfaction, including organisational climate, leadership style, personality traits, and work–family conflict; future research should incorporate these through ministry fixed-effects or instrumental variable designs. Third, the Supervisory Practices mediator and the Job Satisfaction outcome share a source instrument (MSQ-SF). Although Fornell–Larcker and HTMT diagnostics support statistical discriminant validity, residual construct contamination cannot be excluded; future studies should employ a purpose-designed supervisory behaviour scale (e.g., LMX-7; Graen and Uhl-Bien, 1995). Fourth, despite the procedural and post-hoc Common Method Bias controls detailed in Section 3.3, residual common method variance from single-source self-report cannot be excluded. Fifth, EFA and CFA were conducted on the same calibration sample (*N* = 311); external cross-validation on an independent GCC government sample remains a priority. Sixth, H4 was tested with a single MSQ-SF item and is reported as exploratory; replication using a validated multi-item scale (e.g., Effort–Reward Imbalance; Siegrist, 1996) is required. Seventh, the moderation effect sizes (βIN*T* = 0.18 for H6; βIN*T* = −0.14 for H8; IMM = 0.08 and −0.05 for H10) are small to small-to-medium by Cohen’s (1988) conventions; practical recommendations should be treated as hypotheses for pilot validation rather than established prescriptions. Eighth, the four-ministry sample is drawn from central ministries headquartered in Riyadh; generalisability to regional, municipal, or rural government bodies is not established. A formal early-versus-late respondent comparison (Armstrong and Overton, 1977) was not feasible because timestamps were not retained; demographic benchmarking against MHRSD (2024) workforce statistics was conducted instead (see Supplementary material S5). Robustness checks supporting these findings—dummy-coded job-level analysis (Supplementary material S3), JS2-exclusion sensitivity re-analysis (Supplementary material S4), full HTMT ratios (Supplementary material S2), disattenuation-informed correlation estimates (Supplementary material S7), and the extended Fornell–Larcker matrix (Supplementary material S8)—are reported in the Supplementary material, with a methodological-concerns cross-walk provided in Supplementary material S9. Ninth, the Constrained Need Activation (CNA) proposition introduced in Section 5.6 is grounded in a single cross-sectional sample and should therefore be treated as a preliminary, context-specific conceptual interpretation rather than a fully established theoretical framework; its scope conditions, internal consistency, and generalisability remain to be empirically validated through independent replications across GCC and non-GCC public-sector contexts. Five priority directions for future work follow: (i) longitudinal panel designs across Vision 2030 milestones; (ii) marker-variable and correlated-uniqueness CMB diagnostics at the design stage; (iii) multi-item validated equity instruments; (iv) external CFA cross-validation with a purpose-designed supervisory behaviour scale; and (v) regional and municipal extension beyond central-Riyadh ministries.

## 6 Conclusion

This study examined intrinsic and extrinsic motivation as simultaneous predictors of job satisfaction in Saudi public sector bodies (*N* = 311, multi-ministry stratified sample), applying a first-stage moderated mediation framework with gender and job level as simultaneous boundary conditions. To our knowledge, the specific combination of PROCESS Model 7 with gender and job level moderation in Saudi central government has not been previously reported, though related boundary-condition studies exist in the broader GCC context (Aljumah, 2023; AlMarzooqi et al., 2025).

This study advances three principal contributions, tentatively organised within a preliminary, context-specific conceptual proposition we describe as the Constrained Need Activation (CNA) idea, requiring future replication before generalisation. Theoretically, the boundary-collapse proposition holds that Herzberg’s (1966) hygiene–motivator boundary does not hold in this collectivist, high power-distance setting: extrinsic factors carry motivational weight exceeding that of intrinsic factors (*z* = 2.81, *p* = 0.005), and supervisory recognition operates as an active mediating pathway in associational terms rather than a passive contextual variable (H1–H3, H5). The CNA proposition tentatively attributes this collapse to the structural constraint of formal reward channels, which appears to elevate the motivational salience of relational supervisory inputs in this sample. Empirically, the demographic-activation proposition holds that the supervisory mediation pathway is systematically conditioned by demographic boundary positions: gender conditions the intrinsic pathway, with stronger supervisory-mediated satisfaction returns for female employees navigating Vision 2030’s structural role transition (H6, H10); job level conditions the extrinsic pathway, with the strongest returns at entry levels where material need salience is highest (H8, H10). This dual-moderation result extends Self-Determination Theory’s (Deci and Ryan, 2000) relatedness prediction by specifying when relational mediation is most consequential. The exploratory equity test (H4) provides directional support for Adams (1965) prediction as an independent satisfaction determinant, complementing the CNA observations. Methodologically, the simultaneous application of gender and job-level moderation through PROCESS Model 7 in Saudi central government (H10) demonstrates that dual-moderator first-stage moderated mediation yields differentiated HRM prescriptions impossible to derive from single-moderator designs—a methodological refinement applicable to other transitioning workforces undergoing simultaneous gender integration and hierarchical role redefinition (e.g., other GCC states, post-reform Organisation for Economic Co-operation and Development [OECD] ministries)—and provides a preliminary operationalisation of the CNA proposition (pending replication) within Vision 2030’s Human Capability Development Program.

Taken together, these three CNA observations offer a preliminary, demographically disaggregated HRM framework tentatively responsive to the transformative workforce aims of Vision 2030, requiring future replication before broader application. The framework specifies which motivational dynamics need demographic differentiation (intrinsic enrichment for female employees; extrinsic recognition for entry-level staff) and which do not (extrinsic strategies apply equally across gender). This precision is the study’s primary practical value for ministry HRM policy.

Building on the five priority directions outlined in Section 5.7, future research might additionally: (1) replicate across GCC country contexts to establish regional generalisability and distinguish Saudi-specific findings from those generalisable across the broader Gulf region; (2) incorporate Public Service Motivation (Perry and Hondeghem, 2008) as an additional theoretically relevant construct, examining its potential mediating or moderating role in the motivation–satisfaction pathway; (3) conduct qualitative follow-up studies to examine why intrinsic motivation matters more for female employees—the social identity explanation is theoretically plausible but requires empirical validation through interview-based or diary study designs; and (4) test the boundary conditions of the CNA proposition in non-collectivist or low power-distance public-sector contexts to determine whether structural reward constraint alone elevates supervisory recognition as the primary mediating pathway, or whether cultural collectivism is a necessary co-condition.

## Data Availability

The data presented in this study are available on request from the corresponding author. The data are not publicly available due to privacy and ethical restrictions concerning survey participants.
