# Supplementary material for: When hygiene factors become motivation: a moderated mediation analysis of gender, hierarchy, and job satisfaction in Saudi Arabia’s public sector
Source: Front Psychol. 2026 Jun 25;17:1835472. doi: 10.3389/fpsyg.2026.1835472 (PMC13345839; doi:10.3389/fpsyg.2026.1835472)
Supplement: Supplementary file 1 [file Table_1.DOCX]

**Supplementary Material**

*When Hygiene Factors Become Motivation: A Moderated Mediation Analysis of Gender, Hierarchy, and Job Satisfaction in Saudi Arabia’s Public Sector*

Manuscript ID 1835472

*Detailed methodological diagnostics, robustness checks, and supplementary analyses*

This Supplementary Material reports the full technical detail of the methodological diagnostics and robustness checks conducted for the present study. To preserve the readability of the main text, only summary statements and headline figures appear in the manuscript itself; the complete results, computations, and verification analyses are reproduced below. The structure follows the order in which each diagnostic appears in the main text. Each section is self-contained and can be read independently. References are integrated with the main manuscript reference list.

# S1. Common Method Bias Diagnostics

Common Method Bias (CMB) was assessed through design-stage procedural controls and a multi-procedure post-hoc statistical evaluation. The procedural controls (item separation, anonymity assurances, framing of independence from ministry management, and post-hoc construction of interaction terms from mean-centred composites) are described in Section 3.3 of the main text and are not repeated here. This section documents the four post-hoc diagnostics.

## S1.1 Harman's Single-Factor Test

An unrotated principal-components extraction was conducted on all 25 substantive items. The first extracted factor accounted for 29.3% of total variance, well below the conventional 50% threshold beyond which CMB is considered a serious concern (Podsakoff et al., 2003). However, we explicitly acknowledge that Harman's single-factor test has well-documented limitations as a CMB diagnostic for reflective constructs (Podsakoff et al., 2012); accordingly, this result is reported as one of four converging indicators rather than as standalone evidence.

## S1.2 Full Collinearity VIF Assessment

Following Kock (2015), full collinearity Variance Inflation Factors were computed by regressing each construct on all others. All VIF values remained below 3.0, well under the recommended 3.3 threshold for CMB concern. We acknowledge that VIF was not designed specifically for CMB detection and is reported here as a supplementary indicator only.

## S1.3 Variance Heterogeneity of Inter-Construct Correlations

If a single common method factor dominated shared variance, the six inter-construct correlations would converge toward a similar inflated value (Podsakoff et al., 2003). The observed correlations and their dispersion are summarised in Table S1.

***Table S1***

**Variance Heterogeneity Analysis of Inter-Construct Correlations**

| **Construct Pair** | **r** | **Range** | **Interpretation** |
| --- | --- | --- | --- |
| Intrinsic Motivation – Extrinsic Motivation | .33 | lowest | Distinct motivational types |
| Intrinsic Motivation – Supervisory Practices | .48 | — | Moderate |
| Intrinsic Motivation – Job Satisfaction | .53 | — | Moderate-strong |
| Extrinsic Motivation – Supervisory Practices | .57 | — | Moderate-strong |
| Supervisory Practices – Job Satisfaction | .61 | — | Strong (shared MSQ-SF origin) |
| Extrinsic Motivation – Job Satisfaction | .64 | highest | Strong |
| Mean | .53 | — | — |
| SD | .10 | — | Substantial heterogeneity |
| Range | .31 | — | Inconsistent with uniform inflation |

***Note.*** *All correlations significant at p < .001 (two-tailed). The substantial heterogeneity (SD = .10; range = .31) is inconsistent with the convergent inflation that uniform CMB would produce.*

## S1.4 Four-Factor vs. One-Factor CFA Comparison

Following the CFA-based diagnostic recommended by Podsakoff et al. (2003), the theoretically specified four-factor solution was compared with a single-factor alternative. The four-factor solution produced acceptable fit (CFI = .94; TLI = .93; RMSEA = .054 [90% CI: .041, .067]; SRMR = .061), substantially superior to a single-factor solution which would produce CFI ≤ .90 and RMSEA ≥ .10. This pattern is inconsistent with a uniform method-factor explanation.

## S1.5 Acknowledged Limitations of the CMB Approach

Three substantive limitations of this multi-procedure CMB approach are acknowledged. First, no a priori marker variable was embedded in the 25-item instrument; a Lindell–Whitney (2001) marker-variable CFA cannot therefore be retrospectively estimated, since the marker responses do not exist in the dataset. Second, a correlated uniqueness model (Marsh and Bailey, 1991) for same-source items was not estimated. Third, the four-factor vs. one-factor comparison does not model an explicit common method factor. Future replications should: (a) include an a priori theoretically unrelated marker variable; (b) estimate a CFA with an explicit method factor loaded on all items; (c) compare path estimates with and without the method factor; and (d) estimate a correlated uniqueness model for same-source items.

A residual concern specific to the present study is the inter-construct correlation between Supervisory Practices and Job Satisfaction (r = .61), which is consistent with the published supervisory-support literature (Cho and Perry, 2012) but cannot be fully separated from inflation arising from their shared MSQ-SF item origin. This is treated as an explicit limitation in Section 5.7 of the main text, with the recommendation that future research replace the MSQ-SF supervisory items with a purpose-designed supervisory behaviour scale (LMX-7; Graen and Uhl-Bien, 1995).

# S2. Discriminant Validity — Heterotrait–Monotrait (HTMT) Ratio Analysis

Following Henseler et al. (2015), HTMT ratios were computed for all six construct pairs from the full item-level inter-construct correlation matrix using R (psych package; Revelle, 2023). The HTMT ratio is computed as the average of all heterotrait–heteromethod correlations divided by the geometric mean of the average monotrait–heteromethod correlations within each construct.

***Table S2***

**HTMT Ratios for All Six Construct Pairs**

| **Construct Pair** | **HTMT** | **Threshold (.85)** | **Threshold (.90)** |
| --- | --- | --- | --- |
| Intrinsic Motivation – Extrinsic Motivation | .44 | Pass | Pass |
| Intrinsic Motivation – Supervisory Practices | .63 | Pass | Pass |
| Intrinsic Motivation – Job Satisfaction | .64 | Pass | Pass |
| Extrinsic Motivation – Supervisory Practices | .70 | Pass | Pass |
| Extrinsic Motivation – Job Satisfaction | .74 | Pass | Pass |
| Supervisory Practices – Job Satisfaction | .77 | Pass | Pass |

***Note.*** *HTMT ratios computed from the full item-level inter-construct correlation matrix using R (psych package; Revelle, 2023). All six values fall below both the conservative .85 threshold and the liberal .90 threshold (Henseler et al., 2015). The highest value (Supervisory Practices – Job Satisfaction = .77) — the pair flagged as most proximal due to shared MSQ-SF item origin — remains comfortably within acceptable bounds. This supplementary HTMT evidence converges with the Fornell–Larcker assessment reported in the main text (Table 3) to support discriminant validity across all construct pairs.*

# S3. Job Level Moderation — Dummy-Coded Robustness Check

The main analyses treat Job Level as a quasi-continuous moderator (Staff/Specialist = 0; Supervisor/Manager = 1; Director/Senior Manager = 2), following Hayes (2018, p. 249) for ordered categorical moderators with three or more levels. To verify that the moderation conclusions for H8 (supported) and H9 (not supported) are not artefacts of the equal-interval assumption, the moderated regression model was re-estimated using dummy-coded contrasts: D1 (Supervisor/Manager vs. Staff/Specialist) and D2 (Director/Senior Manager vs. Staff/Specialist), with Staff/Specialist as the reference category.

## S3.1 Dummy-Coded Interaction Terms — Extrinsic Motivation (H8)

***Table S3***

**Dummy-Coded Moderation: Extrinsic Motivation × Job Level (H8)**

| **Interaction Term** | **B** | **SE** | **t(298)** | **p** |
| --- | --- | --- | --- | --- |
| EM × D1 (Supervisor vs. Staff) | −.11 | .10 | −1.14 | .257 |
| EM × D2 (Director vs. Staff) | −.29 | .12 | −2.39 | .017 |

***Note.*** *Both coefficients are negative and monotonically ordered (D1 = −.11; D2 = −.29), with no reversal at any hierarchical level. The Director-vs.-Staff contrast (D2) is statistically significant, while the Supervisor-vs.-Staff contrast (D1) is non-significant — confirming that the hierarchical gradient is driven primarily by the Staff–Director difference, consistent with the linear specification reported in the main text.*

## S3.2 Conditional Simple Slopes for Extrinsic Motivation

***Table S4***

**Conditional Simple Slopes — Extrinsic Motivation Predicting Job Satisfaction by Job Level**

| **Job Level** | **B** | **SE** | **t(298)** | **p** | **95% CI** |
| --- | --- | --- | --- | --- | --- |
| Staff/Specialist (0) | .60 | .06 | 9.71 | < .001 | [.48, .72] |
| Supervisor/Manager (1) | .49 | .08 | 6.49 | < .001 | [.34, .63] |
| Director/Senior Manager (2) | .30 | .11 | 2.89 | .004 | [.10, .51] |

***Note.*** *Conditional simple slopes derived from the dummy-coded moderation model. The pattern is monotonically decreasing (Staff > Supervisor > Director) with no reversal, confirming the direction of the H8 effect under both ordinal and dummy-coded specifications.*

## S3.3 Dummy-Coded Interaction Terms — Intrinsic Motivation (H9)

***Table S5***

**Dummy-Coded Moderation: Intrinsic Motivation × Job Level (H9)**

| **Interaction Term** | **B** | **SE** | **t(298)** | **p** |
| --- | --- | --- | --- | --- |
| IM × D1 (Supervisor vs. Staff) | .003 | .10 | 0.03 | .976 |
| IM × D2 (Director vs. Staff) | −.033 | .11 | −0.30 | .767 |

***Note.*** *Both dummy contrasts are negligible and statistically non-significant, confirming that the H9 null finding is invariant to the coding specification. Intrinsic motivation maintains stable predictive strength across all three hierarchical levels.*

## S3.4 Effect Size Estimation — Cohen's Partial f²

Cohen's partial f² for the D2 interaction (Director vs. Staff) on the Extrinsic Motivation pathway = .019, a small effect by Cohen's (1988) conventions (small = .02, medium = .15). This confirms that the practical magnitude of the hierarchical gradient, while statistically detectable, is modest in absolute terms — supporting the cautious framing of the H8-derived practical recommendations in Section 5.5 of the main text.

## S3.5 Conclusion — Coding Specification Robustness

The dummy-coded re-estimation confirms that both H8 (supported) and H9 (not supported) are invariant to the coding specification. The hierarchical gradient pattern is monotonic, the direction and significance of the moderation conclusion is preserved, and the null finding for the intrinsic pathway is stable. The quasi-continuous specification used in the main text PROCESS Model 7 estimation is therefore appropriate for IMM estimation without distorting substantive conclusions.

# S4. Item Content Overlap — JS2-Exclusion Sensitivity Re-Analysis

The Job Satisfaction composite (JS) and the Intrinsic Motivation scale (IM) contain one pair of items with closely related wording: IM3 ("My work is aligned with my personal values and beliefs") and JS2 ("My work is consistent with my personal values and professional goals"). To establish that this content overlap does not materially affect the substantive conclusions, all primary analyses were re-run with JS2 excluded from the Job Satisfaction composite (JS_2item = mean of JS1 and JS3 only).

## S4.1 Reliability of the Reduced Composite

The two-item composite (JS1 + JS3) yields α = .765, computed from CFA-derived inter-item correlations using the Spearman–Brown formula. This remains above the conventional .70 threshold for internal consistency, supporting the use of the reduced composite for sensitivity analysis.

## S4.2 Main-Effects Comparison (H1, H2, H3)

***Table S6***

**Main-Effects Regression — Three-Item vs. Two-Item Job Satisfaction Composite**

| **Predictor** | **β (3-item JS)** | **β (2-item JS)** | **Δβ** |
| --- | --- | --- | --- |
| Intrinsic Motivation | .36 (p < .001) | .32 (p < .001) | .04 |
| Extrinsic Motivation | .52 (p < .001) | .51 (p < .001) | .01 |
| Model R² | .524 | .49 | −.034 |

***Note.*** *Both motivational coefficients remain statistically significant under the reduced composite. The reduction in R² (from .524 to .49) is consistent with the lower internal consistency of the two-item composite (α = .765 vs. .83) and reflects measurement-error attenuation, not a substantive change in the motivation–satisfaction association. H1, H2, and H3 conclusions are unchanged.*

## S4.3 Moderation Pattern Comparison (H6–H9)

***Table S7***

**Moderation Coefficients — Three-Item vs. Two-Item Job Satisfaction Composite**

| **Interaction Term** | **βINT (3-item)** | **p (3-item)** | **βINT (2-item)** | **p** |
| --- | --- | --- | --- | --- |
| Gender × Intrinsic Motivation (H6) | .18 | .003 | .21 | .005 |
| Gender × Extrinsic Motivation (H7) | .07 | .214 | .06 | .239 |
| Job Level × Extrinsic Motivation (H8) | −.14 | .021 | −.14 | .040 |
| Job Level × Intrinsic Motivation (H9) | .06 | .287 | .05 | .301 |

***Note.*** *The significance status of all four moderation hypotheses (H6–H9) is identical across both composite specifications. H6 and H8 remain supported under both; H7 and H9 remain non-significant under both. Direction and approximate magnitude of all coefficients are preserved. The IM3–JS2 wording overlap therefore does not materially affect any moderation conclusion of the study.*

## S4.4 Conclusion — Robustness to Item Overlap

The JS2-exclusion sensitivity analysis provides direct empirical evidence that the substantive conclusions of the study are robust to the IM3–JS2 wording overlap. Main effects, moderation coefficients, and significance status are preserved under the reduced composite specification, with the small reduction in explained variance attributable to measurement-error attenuation rather than substantive change.

# S5. Non-Response Bias — Demographic Benchmarking Analysis

The study achieved a 79.9% response rate (N = 311 of 398 invited). A formal early-versus-late respondent comparison (Armstrong and Overton, 1977) was not feasible because response timestamps were not retained in the anonymised dataset. As an alternative, the obtained sample composition was benchmarked against official population-level workforce statistics for the four participating ministries (MHRSD, 2024).

## S5.1 Gender Composition

***Table S8***

**Sample Gender Composition vs. MHRSD (2024) Workforce Population**

| **Gender** | **Sample (%)** | **MHRSD 2024 (%)** | **Difference** |
| --- | --- | --- | --- |
| Male | 70.1 | ~71 | < 1.0% |
| Female | 29.9 | ~29 | < 1.0% |

***Note.*** *Sample gender distribution falls within one percentage point of the participating ministries' actual workforce composition, providing no evidence of gender-based differential non-response.*

## S5.2 Ministry Sector Composition

***Table S9***

**Sample Ministry Composition vs. Stratified Design Allocation**

| **Ministry Sector** | **Sample (%)** | **Allocation Basis** |
| --- | --- | --- |
| General Administration | 27.0 | Proportional (MHRSD, 2023) |
| Education and Training | 25.4 | Proportional (MHRSD, 2023) |
| Health and Social Affairs | 24.4 | Proportional (MHRSD, 2023) |
| Finance and Economy | 23.2 | Proportional (MHRSD, 2023) |

***Note.*** *Within-stratum non-response was approximately uniform, as proportions match the stratified sampling design with no stratum disproportionately under-represented.*

## S5.3 Job Level Composition

***Table S10***

**Sample Hierarchical Composition vs. Saudi Public Sector Pyramid**

| **Job Level** | **Sample (%)** | **Population Comparison** |
| --- | --- | --- |
| Staff / Specialist | 45.7 | Consistent with pyramidal grading (MHRSD, 2023) |
| Supervisor / Manager | 34.7 | Consistent with pyramidal grading |
| Director / Senior Manager | 19.6 | Progressive scarcity at higher levels |

## S5.4 Worst-Case Bias Estimation

Under a worst-case scenario in which all non-respondents (20.1%) differed from respondents by d = 0.80 SD (a large effect by Cohen's conventions) on all construct means, the implied bias in composite scores would be approximately .16 SD — insufficient to alter the direction or significance of any primary hypothesis test. This bound is conservative; the convergence of demographic benchmarks above suggests the true non-response bias is substantially smaller.

## S5.5 Structural Argument from Differential Findings

A further argument against systematic non-response inflation arises from the pattern of significant (H6, H8) and non-significant (H7, H9) interactions within the same model. A constant additive bias from socially desirable responding cannot selectively elevate two interaction coefficients while leaving adjacent ones at zero; the differential pattern therefore provides structural evidence against a uniform non-response or social-desirability explanation.

## S5.6 Acknowledged Limitations

Three contextual factors mitigate (but do not eliminate) non-response bias concerns: (a) the high response rate (79.9%) leaves limited room for systematic non-response given probabilistic stratified sampling; (b) stratified random sampling from official ministry HRM rosters reduces self-selection effects relative to volunteer recruitment; (c) the anonymisation protocol reduces socially desirable responding relative to name-linked surveys. Future replications should retain timestamped responses to enable Armstrong and Overton (1977) comparisons, and should request HRM distribution officers to confirm in writing that they have no access to individual response data.

# S6. Sensitivity Power Analyses for Null Findings (H7, H9)

To assess whether the null findings for H7 (Gender × Extrinsic Motivation) and H9 (Job Level × Intrinsic Motivation) reflect true absence of moderation rather than insufficient statistical power (Type II error), sensitivity power analyses were conducted in G*Power 3.1 (Faul et al., 2007). Following Hoenig and Heisey (2001), sensitivity analyses were chosen over post-hoc power analyses with observed effect sizes, as the latter is statistically circular and uninformative.

## S6.1 Procedure

Linear multiple regression: F-tests, fixed model, R² increase. Inputs: α = .05 (two-tailed), power = .80, total predictors = 8 (in the full Model 3 with interaction terms), tested predictors = 1 (single interaction term). Output: minimum detectable f² for the interaction term.

## S6.2 Results

***Table S11***

**Sensitivity Power Analysis — Minimum Detectable Interaction Effects**

| **Hypothesis** | **Observed βINT** | **Min. detectable f²** | **Interpretation** |
| --- | --- | --- | --- |
| H7: Gender × Extrinsic | .07 (ns) | 0.037 | Below small-medium threshold |
| H9: Job Level × Intrinsic | .06 (ns) | 0.037 | Below small-medium threshold |

***Note.*** *Minimum detectable f² = 0.037 corresponds approximately to ΔR² ≈ 0.014 — well below conventional benchmarks for small interactions (Cohen, 1988: small = .02). The study was therefore adequately powered to detect any real interaction of small-to-medium magnitude. The null findings for H7 and H9 can be more plausibly interpreted as reflecting true absence of moderation at the population level rather than insufficient statistical power.*

## S6.3 Confidence Interval Evidence

The 95% confidence intervals for the H7 and H9 interaction coefficients further support the true-null interpretation: H7 (Gender × EM): βINT = .07, 95% CI [−.05, .19]; H9 (Job Level × IM): βINT = .06, 95% CI [−.06, .18]. Both intervals are narrow and centred near zero, indicating precise null estimates rather than imprecise non-significant results.

# S7. Disattenuation-Informed Correlation Estimation

To quantify the maximum plausible inflation of the IM–JS correlation attributable to the IM3–JS2 wording overlap, disattenuation-informed correlation estimation was applied. The disattenuated (true-score) correlation between two scales is computed as r_observed divided by the geometric mean of the two scales' reliabilities.

## S7.1 Computations

Three-item Job Satisfaction composite (α = .83) and Intrinsic Motivation (α = .84):

r_true(IM, JS_3item) = .53 / √(.84 × .83) = .53 / .835 = .635

Two-item composite (JS1 + JS3; α = .765) — predicted observed correlation under disattenuation:

r(IM, JS_2item) ≈ .635 × √(.84 × .765) = .635 × .802 = .509

Empirically observed correlation with the two-item composite: r = .51 (within rounding of the disattenuation prediction).

## S7.2 Implication

The convergence between the disattenuation-predicted (.509) and empirically observed (≈.51) two-item correlation indicates that the IM3–JS2 wording overlap contributed at most ~.02 to the original three-item correlation (.53 vs. ~.51 under exclusion). This small magnitude is consistent with the JS2-exclusion sensitivity re-analysis reported in Section S4, and confirms that the substantive conclusions are not artefacts of item-content overlap.

# S8. Fornell–Larcker Discriminant Validity Matrix (Extended)

The Fornell–Larcker (1981) criterion holds that the square root of the Average Variance Extracted (AVE) for each construct should exceed all inter-construct correlations involving that construct.

***Table S12***

**Fornell–Larcker Discriminant Validity — Detailed Comparison**

| **Construct** | **AVE** | **√AVE** | **Largest off-diagonal r** | **Margin** |
| --- | --- | --- | --- | --- |
| Intrinsic Motivation | .53 | .73 | .53 (with JS) | .20 |
| Extrinsic Motivation | .52 | .72 | .64 (with JS) | .08 |
| Supervisory Practices | .50 | .71 | .61 (with JS) | .10 |
| Job Satisfaction | .50 | .71 | .64 (with EM) | .07 |

***Note.*** *All four constructs satisfy the Fornell–Larcker criterion (√AVE > largest inter-construct r). The smallest margin (.07) is between Job Satisfaction and Extrinsic Motivation; the next-smallest (.08) is between Extrinsic Motivation and Job Satisfaction. The Supervisory Practices–Job Satisfaction pair (margin = .10) — flagged as most proximal due to shared MSQ-SF item origin — also satisfies the criterion. Combined with the HTMT evidence in Section S2, discriminant validity is supported across all six construct pairs.*

# S9. Cross-Walk: Methodological Concerns to Supplementary Diagnostics

This section provides a direct mapping between common methodological concerns relevant to cross-sectional moderated mediation research and the supplementary diagnostics conducted in the present study, to facilitate evaluation of the analytical robustness of the reported findings.

***Table S13***

**Methodological Concerns Cross-Walk**

| **Methodological Concern** | **Address in Main Text** | **Supplementary Section** |
| --- | --- | --- |
| Concern 1: Same-sample EFA/CFA | Section 5.7 (limitations) | Section S2 (HTMT) |
| Concern 2: Single-item equity (H4) | Section 4.3, 4.7, 5.7 (exploratory framing) | — |
| Concern 3: CMB diagnostics insufficient | Section 3.3 (transparent acknowledgment) | Section S1 |
| Concern 4: Job level continuous moderator | Section 3.5 (concise rationale) | Section S3 |
| Concern 5: IM3–JS2 item overlap | Section 5.7 (limitation) | Sections S4, S7 |
| Concern 6: Post-hoc power analysis | Section 4.4 (sensitivity reframed) | Section S6 |
| Concern 7: Missing HTMT | Section 5.7 (HTMT < .85) | Section S2 |
| Concern 8: Non-response bias | Section 5.7 (acknowledged) | Section S5 |

# S10. Translation Procedure — Brislin (1970) Four-Stage Forward–Backward Protocol

The 25-item bilingual (Arabic–English) instrument was translated following Brislin’s (1970) systematic forward–backward translation procedure in four stages, designed to ensure semantic equivalence across linguistic and cultural contexts.

**Stage 1 (Forward Translation).** The original English instrument was independently translated into Arabic by two bilingual translators: (a) a certified Arabic–English translator with expertise in organisational psychology terminology, and (b) an academic specialist in Saudi public administration. The two forward translations were produced independently and without consultation between the translators.

**Stage 2 (Reconciliation).** Discrepancies between the two forward translations were systematically reviewed and discussed by the two translators together with a third bilingual researcher acting as facilitator, until consensus was reached on a single reconciled Arabic version.

**Stage 3 (Back-Translation).** A third independent bilingual translator, blind to the original English version, back-translated the reconciled Arabic version into English. This blinding ensures that the back-translation reflects the meaning conveyed by the Arabic version rather than the translator’s knowledge of the original wording.

**Stage 4 (Equivalence Review).** The research team systematically compared the back-translated English version with the original English instrument item by item. No conceptual discrepancies requiring substantive revision were identified across the 25 items. Minor wording adjustments were made to two items based on subsequent pilot feedback (n = 25), with no impact on item content or scale structure.

# S11. Single-Item Equity Operationalisation — Methodological Justification

Hypothesis H4 (perceived effort–reward inequity associated with lower job satisfaction) was tested using a single MSQ-SF item rather than a multi-item Adams equity scale. This section provides the full methodological justification for this operationalisation, the explicit acknowledgement of its limitations, and the rationale for treating H4 as exploratory rather than confirmatory.

**Methodological defensibility.** Single-item operationalisation is defensible for unidimensional, behaviourally specific constructs where (a) the item wording is transparent and unambiguous, and (b) the construct has a singular, concrete referent (Wanous, Reichers, and Hudy, 1997). The effort–reward balance construct meets both criteria: its referent is the perceived fairness of the effort–reward exchange, a concrete and transparent psychological evaluation that does not require multiple indicators to capture its full conceptual domain.

**Structurally independent role.** The effort–reward balance item was deliberately excluded from the three-item Job Satisfaction composite before composite scores were calculated. This separation eliminates part–whole correlation bias that would otherwise arise from regressing a composite-included item against the composite itself, and reflects the structurally independent role given to the equity construct within the analytical framework.

**Acknowledged limitations.** Single-item operationalisation has known psychometric limitations. First, internal consistency reliability cannot be estimated for a single item, so Cronbach’s α is not reported for this indicator. Second, single-item measurement may attenuate observed correlations toward zero when measurement error is uncorrelated with other constructs; however, attenuation toward zero is not guaranteed when measurement error correlates with other constructs (Hoenig and Heisey, 2001). The direction and magnitude of the bias therefore cannot be determined a priori.

**Exploratory framing and replication requirement.** For these reasons, H4 is reported throughout the manuscript as an [Exploratory] hypothesis rather than as a confirmatory test, and is treated as outside the core analytical model (see Section 3.5 of the main text). Substantive theoretical conclusions about Adams’ (1965) equity prediction should not be drawn from the present finding alone. Replication using a validated multi-item equity instrument such as the Effort–Reward Imbalance scale (Siegrist, 1996) is required before the equity–satisfaction association can be characterised with confidence in the Saudi public-sector context.

# S12. Regression Assumption Diagnostics — Detailed Statistics

All standard regression assumptions for the hierarchical moderated regression model were formally tested prior to interpretation of the results. Each diagnostic and its outcome are reported below.

***Table S14***

**Regression Assumption Diagnostic Tests (N = 311)**

| **Assumption** | **Diagnostic Test** | **Statistic** | **Outcome** |
| --- | --- | --- | --- |
| Normality of residuals | Kolmogorov–Smirnov; Q–Q plot inspection | D = .048, p = .08 | Met ✓ |
| Homoscedasticity | Breusch–Pagan test (two motivational predictors) | χ²(2) = 2.31, p = .31 | Met ✓ |
| Multicollinearity | Variance Inflation Factor (all terms) | VIF < 3.0 | Met ✓ |
| Independence of errors | Durbin–Watson | DW = 1.97 | Met ✓ |

*Note.* The non-significant Kolmogorov–Smirnov result indicates no statistically detectable deviation from normality. Furthermore, with N = 311, the Central Limit Theorem renders regression coefficient estimators robust to minor departures from normality even if present. Visual inspection of residual Q–Q plots confirmed approximate normality. The Breusch–Pagan test confirmed homoscedasticity across the two primary motivational predictors. All Variance Inflation Factor values fell well below the conservative 3.0 threshold (Hair et al., 2019), indicating no critical multicollinearity even with the inclusion of mean-centred interaction terms. The Durbin–Watson statistic of 1.97 falls within the 1.5–2.5 range conventionally interpreted as indicating no autocorrelation in residuals.
